# Abundant transcriptomic alterations in the human cerebellum of patients with a *C9orf72* repeat expansion

**DOI:** 10.1007/s00401-024-02720-2

**Published:** 2024-04-19

**Authors:** Evan Udine, Mariely DeJesus-Hernandez, Shulan Tian, Sofia Pereira das Neves, Richard Crook, NiCole A. Finch, Matthew C. Baker, Cyril Pottier, Neill R. Graff-Radford, Bradley F. Boeve, Ronald C. Petersen, David S. Knopman, Keith A. Josephs, Björn Oskarsson, Sandro Da Mesquita, Leonard Petrucelli, Tania F. Gendron, Dennis W. Dickson, Rosa Rademakers, Marka van Blitterswijk

**Affiliations:** 1 Department of Neuroscience, Mayo Clinic, 4500 San Pablo Rd S, Jacksonville, FL 32224 USA; 2 Neuroscience Ph.D. Program, Mayo Clinic Graduate School of Biomedical Sciences, Mayo Clinic, Jacksonville, FL 32224 USA; 3 Department of Quantitative Health Sciences, Mayo Clinic, Rochester, MN 55905 USA; 4 Department of Neurology, Mayo Clinic, Jacksonville, FL 32224 USA; 5 Department of Neurology, Rochester, MN 55905 USA; 6 VIB Center for Molecular Neurology, VIB, Antwerp, Belgium; 7 Department of Biomedical Sciences, University of Antwerp, Antwerp, Belgium

**Keywords:** C9orf72, Amyotrophic lateral sclerosis, Frontotemporal lobar degeneration, Transcriptomics, Cryptic exons

## Abstract

The most prominent genetic cause of both amyotrophic lateral sclerosis (ALS) and frontotemporal lobar degeneration (FTLD) is a repeat expansion in the gene *C9orf72*. Importantly, the transcriptomic consequences of the *C9orf72* repeat expansion remain largely unclear. Here, we used short-read RNA sequencing (RNAseq) to profile the cerebellar transcriptome, detecting alterations in patients with a *C9orf72* repeat expansion. We focused on the cerebellum, since key *C9orf72*-related pathologies are abundant in this neuroanatomical region, yet TDP-43 pathology and neuronal loss are minimal. Consistent with previous work, we showed a reduction in the expression of the *C9orf72* gene and an elevation in homeobox genes, when comparing patients with the expansion to both patients without the *C9orf72* repeat expansion and control subjects. Interestingly, we identified more than 1000 alternative splicing events, including 4 in genes previously associated with ALS and/or FTLD. We also found an increase of cryptic splicing in *C9orf72* patients compared to patients without the expansion and controls. Furthermore, we demonstrated that the expression level of select RNA-binding proteins is associated with cryptic splice junction inclusion. Overall, this study explores the presence of widespread transcriptomic changes in the cerebellum, a region not confounded by severe neurodegeneration, in post-mortem tissue from *C9orf72* patients.

**Supplementary Information:**

The online version contains supplementary material available at 10.1007/s00401-024-02720-2.

## Introduction

A non-coding hexanucleotide repeat expansion in the gene *C9orf72* is the most common cause of both amyotrophic lateral sclerosis (ALS) and frontotemporal lobar degeneration (FTLD) [17, 70]. ALS and FTLD exist on a disease spectrum, characterized by overlap in diagnosis, neuropathology, and genetic risk variants [11, 21, 37, 40, 61]*.* It has been estimated that up to 97% of ALS patients demonstrate TDP-43 pathology, while approximately 50% of FTLD patients have TDP-43 inclusions [5]. Additionally, the *C9orf72* repeat expansion has been shown to contribute to the mis-localization of TDP-43 from the nucleus to the cytoplasm [91], a key feature of TDP-43 pathology. Other studies have demonstrated that expansions in *C9orf72* may lead to disease through at least three main mechanisms: reduced expression of *C9orf72* transcripts, presence of RNA foci, and/or inclusions of dipeptide repeat (DPR) proteins [3, 17, 57]. However, the clinical and pathological variability across the disease spectrum remains largely unexplained.

Recently, the cerebellum has garnered increased attention in the ALS/FTLD research field. Traditionally, the cerebellum, which is clinically unaffected, was thought of as a region primarily spared from TDP-43 pathology and neuronal loss; however, newer studies have shown that TDP-43 protein levels are reduced in this region [63]. In fact, evidence from neuropathological characterization and imaging studies indicate that the cerebellum is involved in both ALS and FTLD [66, 73]. Furthermore, *C9orf72* RNA foci and DPRs are widely observed throughout the cerebellum [26, 27], and features of the *C9orf72* repeat expansion have been associated with clinico-pathological variables of the disease, specifically in this region [16, 71, 83]. Some studies have previously been completed to evaluate transcriptomic alterations in the cerebellum across the ALS/FTLD disease spectrum. For example, using a gene expression assay, we described a cerebellar upregulation of homeobox (*HOX*) genes in patients with a *C9orf72* repeat expansion [24]. Additional studies have used RNA sequencing (RNAseq) to identify transcriptome-wide differentially expressed genes and alternative splicing events. One such study compared ALS patients with the repeat expansion to sporadic ALS subjects and controls and found pervasive gene expression changes in the cerebellum [67]. In this region, differentially expressed genes were involved in neuron development, protein localization, and transcription pathways [67]. They also discovered widespread splicing dysregulation, specifically alternative splicing of cassette exons. The genes that contained these alternative splicing events were primarily involved in cell signaling, cellular trafficking, synaptic function, and RNA processing [67]. Despite these efforts, studies of the cerebellar transcriptome have been limited by sample size and/or the computational tools to evaluate splicing that were available at the time.

Regardless, transcriptomic and functional studies have consistently demonstrated that alternative splicing and dysregulation of RNA splicing are key drivers of the ALS/FTLD disease spectrum [2, 13, 35, 44, 65, 79, 88, 92]. In fact, these studies have not only identified dysregulation of cassette exon splicing, but also the presence of cryptic exons in ALS/FTLD patients with TDP-43 pathology [51, 67]. Cryptic exons can be defined as exons that are present within a normally intronic region, often leading to a loss of expression, possibly by nonsense mediated decay, or the production of a dysfunctional protein. These studies have shown that nuclear, endogenous TDP-43 binds to and represses these cryptic exons [51]. One example of a cryptic exon that can be present in the brain of FTLD patients with TDP-43 pathology is in the gene *STMN2*, where loss of TDP-43 fails to suppress a cryptic exon, leading to a decrease in both RNA and protein levels and an increase in truncated *STMN2* transcripts [41, 56]. This has been observed in patient tissue, allowing *STMN2* to serve as a marker for this pathology [41, 56, 68]. Moreover, 2 studies have indicated that inclusion of a cryptic exon in *UNC13A* confers disease risk, where the cryptic exon is found in the absence of nuclear, endogenous TDP-43 [10, 52].

In summary, the cerebellum exhibits ample *C9orf72* pathology, but minimal neurodegeneration in autopsy tissue, making it a prime region for post-mortem study of *C9orf72* transcriptomics. This gives us the opportunity to uncover findings that may have remained hidden when focusing on primary affected regions with extensive neuronal loss and that could hint at mechanisms underlying the resilience of the cerebellum to neurodegeneration despite evidence of TDP-43 dysfunction. Therefore, to capture genes, transcripts, or pathways that may contribute to the heterogeneity of the ALS/FTLD disease spectrum, here we profiled the cerebellar transcriptome of patients with or without a *C9orf72* repeat expansion, as well as controls. We included subjects for whom post-mortem tissue was available through the Mayo Clinic Brain Bank. Using differential gene expression, co-expression network analysis, and differential splicing, we aimed to determine expression and splicing alterations in this disease spectrum.

## Methods

### Cohort description

In total, we performed RNAseq on cerebellar tissue from 193 samples: 60 patients who harbored a *C9orf72* repeat expansion (c9), 108 patients who did not harbor a *C9orf72* repeat expansion and displayed TDP-43 pathology (Types A and B; non-c9), and 25 controls with no evidence of neurological diseases (control). Samples were provided by the Mayo Clinic Brain Bank and selected based on a neuropathological diagnosis of ALS (*n* = 56), FTLD-TDP (*n *= 67), FTLD-TDP with ALS (FTLD-ALS; *n* = 43), c9 patients with other diagnoses (Alzheimer’s disease and depressive pseudodementia; *n* = 2) [9], and control subjects (*n* = 25). For c9 patients, the median age at death was 66 years [interquartile range (IQR): 61–73], median RNA integrity number (RIN) value was 9.1 (IQR: 8.7–9.5), and 43% were female. For non-c9 patients, the median age at death was 72 years (IQR: 64–83), median RIN value was 9.4 (IQR: 8.9–9.7), and 44% were female. For controls, the median age at death was 87 years (IQR: 80–89), median RIN value was 9.2 (IQR: 8.5–9.6), and 52% were female. See Table 1 for further information.

**Table 1:** Cohort characteristics

	**c9 (*n* = 60)**	**non-c9 (*n* = 108)**	**control (*n* = 25)**
Age at death (IQR)	66 (61–73)	72 (64–83)	87 (80–89)
RIN (IQR)	9.1 (8.7–9.5)	9.4 (8.9–9.7)	9.2 (8.5–9.6)
Sex (Female)	26 (43%)	47 (44%)	13 (52%)
Diagnosis (FTLD)	23 (38%)	44 (41%)	0 (0%)
Diagnosis (FTLD–ALS)	16 (27%)	27 (25%)	0 (0%)
Diagnosis (ALS)	19 (32%)	37 (34%)	0 (0%)
Diagnosis (Other)	2 (3%)	0 (0%)	0 (0%)

Overview of information for all samples. We included a total of 193 samples, including 60 patients with a pathological diagnosis of frontotemporal lobar degeneration (FTLD), amyotrophic lateral sclerosis (ALS), frontotemporal lobar degeneration with amyotrophic lateral sclerosis (FTLD-ALS), or 2 other patients including one with Alzheimer’s disease and one with pseudodementia who had a *C9orf72* repeat expansion (c9), 108 patients with FTLD, FTLD-ALS, or ALS who did not have a *C9orf72* repeat expansion (non-c9), and 25 control subjects with no known neurological disorders (control). Age at death, RIN, sex, and primary pathological diagnosis is included. The median values are shown for age at death and RIN, and interquartile range (IQR). The frequency and percentage of female participants are shown for sex, and the frequency and percentage are shown for the diagnosis group

### RNA sequencing

RNA was extracted from frozen cerebellar brain tissue (lateral cerebellar cortex) with the RNAeasy Plus Kit (Qiagen) using on-column DNase treatment. The RNA concentration was calculated using NanoDrop absorbance (Thermo Scientific). RNA quality was assessed with the 2100 Bioanalyzer Instrument (Agilent), utilizing the RNA Nano chip (Agilent) to calculate RIN values. Samples were only included for sequencing with a RIN value ≥ 6.0. Libraries were prepared with the TruSeq RNA Library Prep Kit (Illumina) and paired-end sequencing was completed using the HiSeq 4000 (Illumina) with 10 samples/lane and 101 base-pairs per read at Mayo Clinic’s Genome Analysis Core. Raw RNAseq reads were analyzed with MAP-Rseq (v3.1.3) [38]. Briefly, reads were aligned to the reference human genome (hg38) with a splice-aware STAR aligner (v2.5.2c) [20]. Gene and exon expression quantification were performed using Subread (v1.5.1) [49] to obtain raw read counts and calculate reads per kilobase million (RPKM).

### Quality control

We completed extensive quality control. On a per sample level, we evaluated RIN, number of total sequencing reads, mapped reads, gene body coverage, and GC content. Additionally, we performed principal component analysis (PCA) and confirmed the reported sex based on expression of genes on the X and Y chromosomes. Notably, all 193 samples met our quality control metrics.

### Data normalization

Gene expression data were normalized using conditional quantile normalization (CQN) [32]. CQN is a method to normalize RNAseq datasets that considers gene counts, gene length, and GC content. Not expressed and lowly expressed genes were removed (maximum normalized and log2-transformed RPKM values less than 2.5), giving us a total of 25,928 genes for downstream analyses.

### Differential expression and deconvolution

Prior to differential expression analysis, we performed source of variation (SOV) analysis using linear regression and subsequently an ANOVA, extracting the F-statistic. Variables with a mean F-statistic above 1.4 were included in downstream analyses as covariates. We used 2 different linear models for differential expression. The first model included sex, gene count, RIN, age, plate, and disease group (c9, non-c9, and control), while the other model also included markers for the 5 major brain cell types (neurons, microglia, astrocytes, oligodendrocytes, and endothelial cells), as determined by using 50 randomly selected genes using the R package BRETIGEA (Online Resource Table 1a) [55]. Fold-changes were calculated and *P* Values were adjusted for multiple testing using a false discovery rate (FDR) correction. We considered genes with an FDR less than 5% to be statistically significant (FDR < 0.05). We used ggplot for volcano plots, boxplots and barcharts, the VennDiagram package to create Venn diagrams, leafviz to display LeafCutter sashimi plots, and the ComplexHeatmap [30] and flashClust packages with the Euclidean distance and average method to generate heatmaps. For our deconvolution analysis, proportions of 5 major brain cell types were estimated using the same BRETIGEA markers (Online Resource Table 1a). Cell proportion differences were evaluated using a Kruskal–Wallis rank-sum test and pairwise comparisons were assessed with a Wilcoxon rank-sum test.

### Immunofluorescence staining

To further investigate our deconvolution findings related to oligodendrocytes, we quantified the number of OLIG2+ cells, a marker for oligodendrocyte cell lineage [36, 39, 50, 60], on 12 samples. Immunofluorescence was performed on paraffin‐embedded cerebellar human tissue. In brief, the paraffin blocks were sliced into 5‐μM thick sections, deparaffinized with xylene and rehydrated with decreasing concentrations of ethanol. Sections were then incubated with primary antibodies (Anti-OLIG2, 1:25, cat# ab109186, Abcam) at 4 °C overnight in a humid chamber. After washing with 1X phosphate buffered saline (PBS), sections were incubated for 1 h with an Alexa Fluor (594)-conjugated secondary antibody (goat anti-rabbit, 1:500, cat# A11012, Invitrogen) at room temperature, washed with 1X PBS, treated with 0.2% Sudan Black B in 70% ethanol for 10 min and washed 3 times with 1X PBS, before being cover-slipped and mounted using ProLong® Gold Anti-fade Reagent with DAPI (cat# P36935, Invitrogen). Images were captured using a confocal microscope (LSM880, Zeiss), and 3 tiled images (4 × 4) were taken for each sample, using the 20 × objective. Cell numbers were counted in the white matter using Cell Counter from FIJI and normalized for the total white matter area, using the region of interest (ROI) tool. Two independent, blinded investigators performed cell counting. Statistical analysis was completed using the mean of the 3 ROIs per sample and then calculating Spearman’s correlation coefficient *r* between the counts for the 2 investigators.

### Co-expression analysis

Additionally, we identified modules of correlated genes to prioritize relevant biological networks that may differ between disease groups. For module identification, we utilized weighted gene co-expression network analysis (WGCNA) [45]. We completed 1 analysis for each of the 3 pairwise comparisons (c9 vs. control, c9 vs. non-c9, and non-c9 vs. control) using residual expression values calculated from the previously described models. Modules were constructed using a signed hybrid network and biweight mid-correlation (“bicor”) with a power threshold of 6 to maintain a scale-free topology. We used the dynamic tree cutting method with a merge height of 0.4 and a minimum module size of 30. These settings were consistent in all 3 analyses. We calculated module eigengene by the first principal component and assigned each module a unique color. Module membership (MM) was determined by correlating the expression of all genes with each module’s eigengene value. Network visualization was completed using cytoscape (v 3.8.2) [74]. Networks included all genes with a MM of > 0.6, which were visualized using the force-directed yFiles Organic Layout and Organic Edge Router algorithms in cytoscape. The node size represents the gene connectivity, the color represents the assigned module, and the edge thickness represents the correlation effect size. Module preservation analysis was completed using the modulePreservation function from the WGCNA R package.

### Splicing

To detect differentially spliced genes between conditions, LeafCutter (v0.2.6) [48], which identifies variable intron splicing from short reads and finds alternate splicing of high complexity, was used. Intron clusters were defined to be differentially spliced if they had an FDR < 0.05 and at least one intron excision event with a change in percent spliced in (Δ PSI) of 10% in either direction ( >|10%|). Visualization of splice junctions was performed using the R package leafviz, which is available as a part of the LeafCutter repository. In addition to the standard differential splicing comparisons, we looked specifically at cassette exons and cryptic splicing events. We identified cassette exons by using a cassette exon classification script that is part of the LeafCutter GitHub repository. We calculated percent inclusion of cassette exons as previously used by others in the field [7]. For cryptic splicing analysis, we utilized 2 different strategies. First, as others have argued that cryptic exons should not rely solely on annotations, as these continue to change and improve, one analysis was focused on the percent inclusion of cassette exons. Events identified in this analysis are referred to as cryptic cassette exons [7]. We considered cryptic cassette exons to be those that are present in less than 10% of controls (or non-c9 subjects in the c9 vs. non-c9 analysis) and have a Δ PSI > 10%, and skiptic cassette exons, which are constitutively included exons that are skipped, to be present in more than 90% of controls (or non-c9 subjects in the c9 vs. non-c9 analysis) with a Δ PSI < -10%. Second, we completed a cryptic splicing analysis based on the LeafCutter assignment. To discover these cryptic splicing events, we identified differentially spliced clusters that contained cryptic three prime, cryptic five prime, or cryptic unanchored splice junctions with a Δ PSI >|10%| for that specific event (junction).

### cDNA sequencing

cDNA, prepared from DNase-treated RNA of 9 individuals, was amplified and sequenced to confirm the presence of novel cryptic cassette exons. First-strand cDNA was obtained with the Superscript III system (Invitrogen) using oligo-(dT), random hexamers and gene specific primers (MAP4K3:5′-GTAATATAGCCACACCCAAG-3′/AGL:5′-CCAAACTATGGATTTCTAGGC-3′) designed towards the 3' end of the respective genes. To amplify specifically spliced products, PCR primers were designed within the cryptic exon and both upstream and downstream exons for each gene. To ensure cDNA specific amplification, all the non-cryptic exon PCR primers were designed to span exon boundaries, with a genomic DNA sample also included as a negative control for all reactions. The purified products were sequenced, in both directions, on the ABI 3730 platform using BigDye Terminator cycle sequencing reagents (Applied Biosystems).

### Long-read RNAseq

We completed Pacific Biosciences (PacBio) long-read RNAseq (Iso-Seq) on 2 samples. For this, RNA was extracted from frozen brain tissue (cerebellum), using a TRIzol and chloroform precipitation followed by the RNAeasy Plus Kit with on-column DNase treatment. RNA concentration was calculated using NanoDrop absorbance. RNA quality was assessed using the 2100 Bioanalyzer Instrument with the RNA Nano chip. Samples were only included for sequencing with a RIN value ≥ 6.0. Libraries were prepared following the standard Iso-Seq protocol at Mayo Clinic’s Genome Analysis Core. Circular consensus sequencing (ccs) occurred on the Sequel II, multiplexing 2 samples per SMRT cell. For analysis, ccs reads were generated from subreads using the package pbccs (v6.4.0) and samples were demultiplexed using cDNA cupcake (v24.3.0). We performed various levels of quality control using the package isoseq3 (v3.4.0). Transcripts of interest were visualized using the Integrative Genomics Viewer (IGV [v2.16.]).

### Pathway analysis

To examine whether specific gene sets were enriched for biological processes and pathways, enrichment analysis was performed using the anRichment package with gene sets from the molecular signatures database (MsigDB; v7.4) [78].

### Additional statistical analyses

For other analyses, Pearson’s correlation coefficient *r* was obtained using the rcorr function from the package Hmisc (v5.1.0) in R (v4.3.0). Bonferroni-adjusted *P* Values were calculated using the p.adjust function in R, adjusting for all tests completed (number of splicing events within the comparison group multiplied by the number of genes encoding for RNA-binding proteins; 2592 tests for the c9 vs. control annotation-based analysis, 256 tests for the c9 vs. non-c9 annotation-based analysis, 256 tests for the non-c9 vs. control annotation-based analysis).

## Results

### Gene expression

**Fig. 1:**
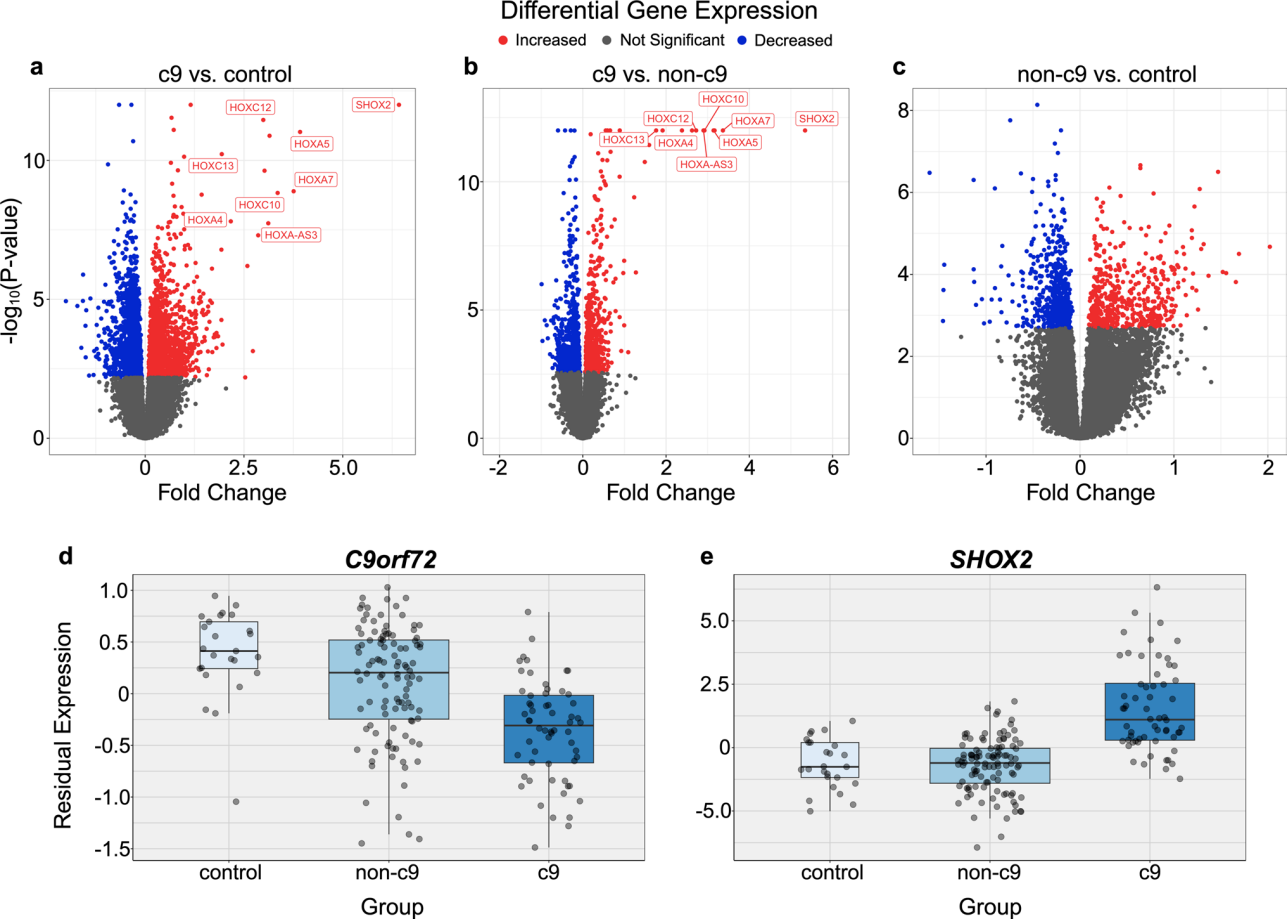
Differential expression analyses. **a**–**c** Volcano plot of differentially expressed genes. **a** *C9orf72* expansion patients compared to controls (c9 vs. control). **b** *C9orf72* expansion patients compared to non-*C9orf72* expansion patients (c9 vs. non-c9) **c**, and non-*C9orf72* expansion patients compared to controls (non-c9 vs. control). Significantly differentially expressed (FDR < 0.05) homeobox genes are displayed. The *x*-axis shows fold-changes and the *y*-axis -log_10_ of *P* Values. Fold-changes are scaled to 0 for visualization purposes. **d**, **e** Boxplots of 2 differentially expressed genes including **d** *C9orf72* (FDR = 3.65E-09) and **e** *SHOX2* (FDR = 2.15E-21). Residual expression including markers for cell types are displayed. Boxplots represent the median with interquartile range (IQR)

We performed RNAseq on 193 subjects. Overall, no outliers were detected, and samples did not separate by group using PCA (Online Resource Fig. 1a). When using a linear regression model that accounted for sex, gene count, RIN, age, plate, and differences in cellular proportions, we identified 6911 differentially expressed genes (FDR < 0.05) among groups (Online Resource Table 2). Of those genes, 12 have previously been associated with ALS and/or FTLD, including *C9orf72* (FDR = 3.65E-09; Fig. 1d), *CDH22* (FDR = 2.28E-05), *TARDBP* (FDR = 0.011), *CAMTA1* (FDR = 0.034), *FUS* (FDR = 0.042), and *OPTN* (FDR = 0.046).

Additionally, we completed 3 pairwise differential expression analyses, which included patients with a *C9orf72* repeat expansion and controls (c9 vs. control), patients with a *C9orf72* repeat expansion and patients without a *C9orf72* repeat expansion (c9 vs. non-c9), and patients without a *C9orf72* repeat expansion and controls (non-c9 vs. control). We found 3383 differentially expressed genes in the c9 vs. control analysis (Fig. 1a, Online Resource Fig. 1b, Online Resource Table 2), 1369 differentially expressed genes in the c9 vs. non-c9 analysis (Fig. 1b, Online Resource Fig. 1b, Online Resource Table 2) and 1037 differentially expressed genes in the non-c9 vs. control analysis (Fig. 1c, Online Resource Fig. 1b, Online Resource Table 2).

Using the top 50 differentially expressed genes from each pairwise comparison, we performed pathway enrichment analysis and found that in the c9 vs. both non-c9 and control, differentially expressed genes were enriched for pattern specification and regionalization (Online Resource Table 3). Genes were not enriched for any biological processes in the non-c9 vs. control comparison (Online Resource Table 3). Our findings align with a previously reported upregulation of *HOX* genes in the c9 subjects compared to both non-c9 and control subjects [24]. Notably, the gene *SHOX2* (FDR = 2.15E-21) was one of the top differentially expressed genes in all models tested (Fig. 1a, b, e).

To identify important similarities and differences between brain regions, we then compared these differential expression results to our previously published frontal cortex study [19]. In total, 1957 of the 6911 (28%) differentially expressed genes overlapped between these two regions (Online Resource Fig. 1d), including *C9orf72*, *TARDBP*, *CAMTA1*, *FUS*, and *OPTN*. We did notice that *HOX* genes were detected only in the cerebellum, such as *SHOX2*, *HOXC12*, *HOXA5*, and *HOXC13*.

Hereafter, we compared cellular proportions using marker genes selected for 5 major cell types in the brain (neurons, microglia, astrocytes, oligodendrocytes, and endothelial cells) that were included in differential expression analyses. We detected no significant changes in neurons, microglia, astrocytes, or endothelial cells in c9 patients compared to non-c9 patients or controls (Online Resource Fig. 2a). We did, however, find an overall change in the estimated proportion of oligodendrocytes between groups (e.g., c9, non-c9, and control, *P* Value = 6.74E-06). When examining each of the 3 pairwise comparisons, we noticed that there was a decrease in the proportion of oligodendrocytes in the c9 group compared to both non-c9 (*P* Value = 1.80E-05; Online Resource Fig. 2a) and control groups (*P* Value = 7.52E-05; Online Resource Fig. 2a). To confirm this reduction, we performed an exploratory immunostaining experiment using oligodendrocyte lineage cell marker OLIG2 [36, 39, 50, 60] in 12 cerebellar samples (Online Resource Fig. 2b). We selected 6 samples with a relatively high proportion of oligodendrocytes, and 6 with a fairly low proportion of oligodendrocytes, as suggested by our deconvolution analysis. Our exploratory experiment revealed that the number of OLIG2 + cells was significantly increased in the high group compared to the low group (*P* Value = 0.041; Online Resource Fig. 2c).

### Co-expression analysis

Next, we looked to identify modules of correlated genes that may differ between disease groups using co-expression analysis. In the c9 vs. control analysis, we identified 24 total modules (Online Resource Fig. 3a, Online Resource Table 4a). Of these 24 modules, 7 were significantly associated with disease group (c9; Table 2). Pathway analysis of the downregulated disease-associated modules revealed involvement in small molecule metabolic processes (Blue; Online Resource Fig. 4a–b, Table 2) and mitochondrion organization (Yellow), while the upregulated disease-associated modules were involved in pattern specification (DarkRed; Online Resource Fig. 4c), protein localization (Turquoise), protein modification (Brown), nucleosome assembly (DarkGreen), and defense response (MidnightBlue; Table 2). Interestingly, the Blue module, enriched for genes involved in metabolic processes, included *C9orf72* as a hub gene, similar to a module we observed in the frontal cortex [19]. Furthermore, the DarkRed module (Online Resource Fig. 4c), enriched for genes in pattern specification (Table 2), contained many of the *HOX* family genes that were differentially expressed (e.g., *SHOX2*)*.* We performed PCA using the residual expression of genes belonging to this module and found separation of c9 samples and a subset of non-c9 samples (Online Resource Fig. 4d).

**Table 2:** Disease-associated modules

Analysis	Module	Direction	Pathway (GO:BP)	FDR
c9 vs. control	Blue	Down	Small molecule metabolic process	2.02E-12
	Yellow	Down	Mitochondrion organization	4.70E-12
	DarkRed	Up	Pattern specification	3.99E-12
	Turquoise	Up	Protein localization	7.41E-04
	Brown	Up	Protein modification	2.57E-06
	DarkGreen	Up	Nucleosome assembly	3.99E-12
	MidnightBlue	Up	Defense response	1.96E-24
c9 vs. non-c9	Pink	Down	RNA localization	6.44E-03
	Yellow	Down	Regulation apoptosis	2.00E-02
	LightGreen	Down	Vasculature development	1.54E-22
	Salmon	Down	Ensheathment of neurons	1.71E-11
	DarkTurquoise	Down	Actin filament organization	8.43E-03
	Green	Down	Nervous system process	4.04E-10
	DarkRed	Up	Pattern specification	9.06E-12
	Orange	Up	Chromatin organization	7.74E-10
	RoyalBlue	Up	mRNA processing	7.76E-06

Sixteen modules were associated with disease group (c9 status) when comparing *C9orf72* expansion patients to control samples (c9 vs. control) and when comparing *C9orf72* expansion patients to non-*C9orf72* expansion patients (c9 vs. non-c9). The Table shows (left to right) the analysis in which the module was identified, the module color, the direction of effect, the associated module pathway, and the enrichment analysis false discovery rate (FDR)

In the c9 vs. non-c9 analysis, we identified 25 modules (Online Resource Fig. 3b, Online Resource Table 4b). Of these 25 modules, 9 were significantly associated with the c9 disease group (c9; Table 2). Pathway analysis showed that the downregulated disease-associated modules were involved in RNA localization (Pink), regulation of apoptosis (Yellow), vasculature development (LightGreen), neuron ensheathment (Salmon), actin filament organization (DarkTurquiose), and nervous system process (Green), while the upregulated disease-associated modules were involved in pattern specification (DarkRed), chromatin organization (Orange), and mRNA processing (RoyalBlue; Table 2). The DarkRed module was similar to what was shown in the c9 vs. control analysis, also containing *SHOX2* and many other *HOX* family genes.

In the non-c9 vs. control analysis, we identified 21 modules (Online Resource Fig. 3c, Online Resource Table 4c). Of these 21 modules, 5 were significantly associated with the disease group (non-c9). Pathway analysis showed that the downregulated disease-associated modules were involved in small molecule metabolic processes (Blue) and synaptic signaling (Orange), while the upregulated disease-associated modules were involved in vasculature development (MidnightBlue), RNA binding (Turquoise), and mRNA processing (Brown).

Our enrichment results highlighted the importance of metabolic, mRNA, and protein processing pathways as relevant dysregulated processes. More specifically, when focusing on c9-associated modules (7 c9 vs. control, 9 c9 vs. non-c9), 7 contained multiple known disease-associated genes. For example, in the c9 vs. control analysis, the Blue module (downregulated; Online Resource Fig. 3a–b, Online Resource Table 4a) contained 19 disease-associated genes, including *C9orf72, NEK1, FUS, TBK1,* and *TMEM106B.* Additionally, the Brown module (upregulated) contained 12 disease-associated genes, including *EPHA4*, *GRN*, *MAPT*, *OPTN*, *CHMP2B*, and *UNC13A.* Moreover, in the c9 vs. non-c9 analysis, the RoyalBlue module (upregulated) contained 7 disease-associated genes, some of which include *MAPT*, *OPTN*, *SOD1*, and *TARDBP*, emphasizing the involvement of known disease-associated genes in these modules (Online Resource Tables 4a, b).

Furthermore, we performed a module preservation analysis between the modules identified in the current study and those from our previous frontal cortex study [19]. We considered a module preservation score > 10 an indication of preservation. There was substantial preservation of cerebellar modules in the frontal cortex across all 3 pairwise analyses (Online Resource Fig. 5a–c). In fact, most cerebellar modules were preserved in the frontal cortex. As an example, we noticed several c9-associated modules that were strongly preserved, including the Blue module involved in small molecule metabolic processes and the Brown module involved in protein modification. Notably, the DarkRed module enriched for pattern specification was not preserved (Online Resource Fig. 5a, b).

### Differential splicing

**Fig. 2:**
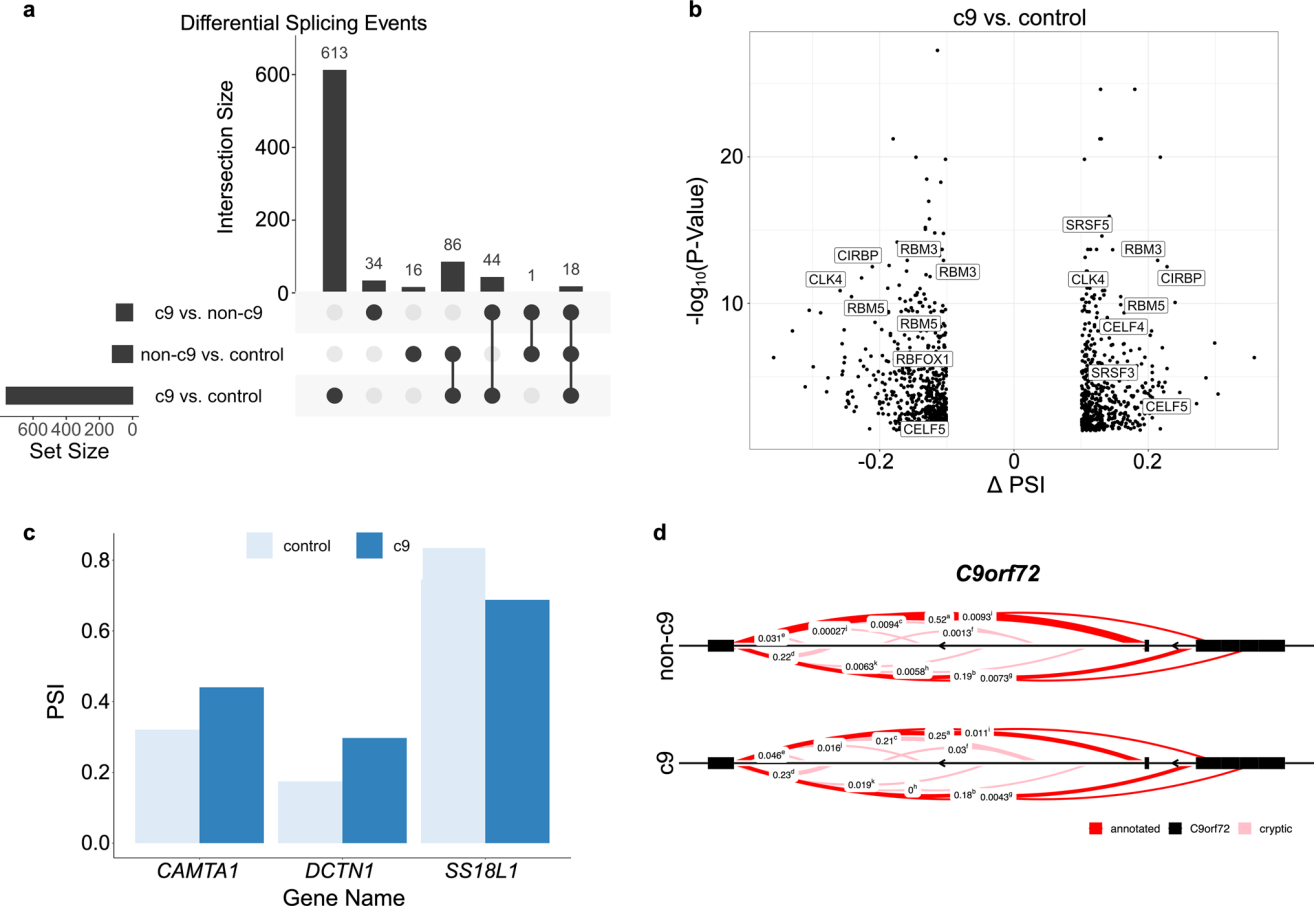
Differential splicing analysis. **a** Upset plot to demonstrate number of genes that contain at least one differential splicing event for each comparison. **b** Volcano plot of differentially spliced intron junctions (FDR < 0.05) for *C9orf72* expansion patients (c9) compared to controls (c9 vs. control). The *x*-axis shows the change in percent spliced in (Δ PSI) and the *y*-axis shows -log_10_ of the *P* Value. Genes that contain differentially spliced clusters in significantly enriched pathways are displayed. **c** Barplot of PSI for the 3 differentially spliced ALS/FTLD associated genes in the c9 vs. control analysis. **d** Sashimi plot of the cryptic splicing event in *C9orf72* for c9 patients and non-*C9orf72* expansion patients (non-c9). Pink lines represent cryptic splice junctions as determined by LeafCutter and solid red lines represent annotated splice junctions. Numbers represent the average inclusion for that splice junction

We used LeafCutter to perform differential splicing analysis. We found 815 differentially spliced intron clusters (FDR < 0.05, Δ PSI >|10%|) corresponding to 761 unique genes in the c9 vs. control comparison, 101 differentially spliced intron clusters corresponding to 97 unique genes in the c9 vs. non-c9 comparison, and 123 differentially spliced intron clusters corresponding to 121 unique genes in the non-c9 vs. control comparison (Fig. 2a, Online Resource Tables 5a–c). We first looked at differential splicing of disease-associated genes. We observed 4 differential splicing events in these genes (Fig. 2c, d). This includes an exon skipping event in *CAMTA1* (c9 vs. control, FDR = 5.50E-06; Fig. 2c, Online Resource Fig. 6a), an exon skipping event in *DCTN1* (c9 vs. control, FDR = 1.32E-04; non-c9 vs. control, FDR = 0.002; Fig. 2c, Online Resource Fig. 6b), increased inclusion of an annotated exon in *SS18L1* (c9 vs. control, FDR = 6.55E-05; Fig. 2c, Online Resource Fig. 6c), and a complex cryptic splicing event in *C9orf72* (c9 vs. non-c9, FDR = 7.31E-10; Fig. 2d, Online Resource Tables 5a–c). We completed pathway enrichment analysis using all genes that contained a differential splicing event from each comparison. In the c9 vs. control analysis, we found enrichment for pathways involved in regulation of RNA splicing (Fig. 2b, Online Resource Table 6). No significant enrichments were present in the other 2 pairwise comparisons (Online Resource Table 6). In general, there did not appear to be a unifying feature among differential splicing events (e.g., based on intron length, gene length, location within gene, and GC content).

**Fig. 3:**
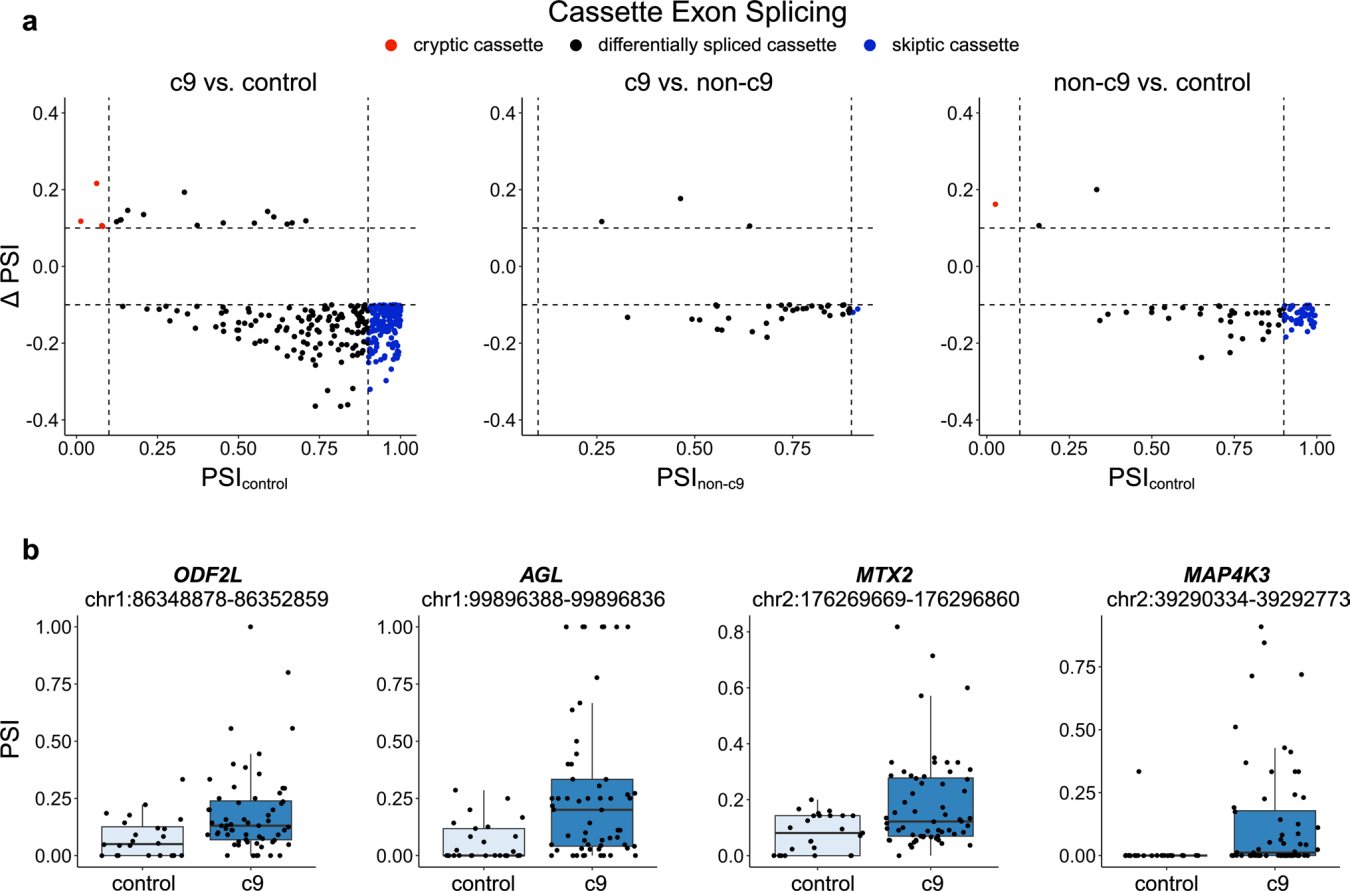
Cassette exon differential splicing. **a** Scatter plots of differentially spliced cassette exons. *Left:* Scatter plot displaying the percent spliced in (PSI) for control samples on the *x*-axis and change in PSI (Δ PSI) on the *y*-axis comparing *C9orf72* expansion patients (c9) to controls (c9 vs. control). *Middle:* Scatter plot displaying the PSI for non-*C9orf72* expansion patients (non-c9) on the *x*-axis and Δ PSI on the *y*-axis comparing c9 patients vs. non-c9 patients (c9 vs. non-c9). *Right:* Scatter plot displaying the PSI for control samples on the *x*-axis and Δ PSI on the *y*-axis comparing non-c9 patients to controls (non-c9 vs. control). Cryptic cassette exons are colored red and skiptic cassette exons are colored blue. **b** Boxplots of cassette exon percent inclusion for the 4 cryptic cassette exons identified in the c9 vs. control analysis, which includes *ODF2L* (FDR = 0.028), *AGL* (FDR = 0.002), *MTX2* (FDR = 0.002), and *MAP4K3* (FDR = 1.39E-06). Boxplots represent the median with interquartile range (IQR)

Next, we looked specifically at alternative splicing of cassette exons, as skipping of these exons has been shown to be dysregulated previously in the cerebellum of c9 patients [67]. We calculated percent inclusion of the cassette exons as described elsewhere [7]. Using our differential splicing criteria (FDR < 0.05, Δ PSI >|10%|), we found 303 differentially spliced cassette exons in the c9 vs. control analysis, 37 differentially spliced cassette exons in the c9 vs. non-c9 analysis, and 81 differentially spliced cassette exons in the non-c9 vs. control analysis (Fig. 3a, Online Resource Table 7a–c). Similar to previous reports, there was a relatively high number of exon skipping events in c9 patients, with 285 skipped cassette exons in the c9 vs. control analysis (94% of differentially spliced cassette exons) and 34 in the c9 vs. non-c9 analysis (92% of differentially spliced cassette exons).

### Cryptic splicing

We were particularly interested in cryptic splicing, given evidence of its dysregulation across the ALS/FTLD disease spectrum [28, 41, 51, 52, 56, 68]. We classified cryptic splicing events using 2 different methods (see methods for further details). First, we classified cryptic and skiptic cassette exons considering the PSI. Using these criteria for the cassette exon analysis, we revealed 4 cryptic cassette exons and 157 skiptic cassette exons in c9 patients and 1 cryptic cassette exon and 44 skiptic cassette exons in non-c9 patients (Fig. 3a) in comparison to controls. The 4 cryptic cassette exons in c9 patients were in the genes *ODF2L* (FDR = 0.028), *AGL* (FDR = 0.002), *MTX2* (FDR = 0.002), and *MAP4K3* (FDR = 1.39E-06; Fig. 3b).

**Fig. 4:**
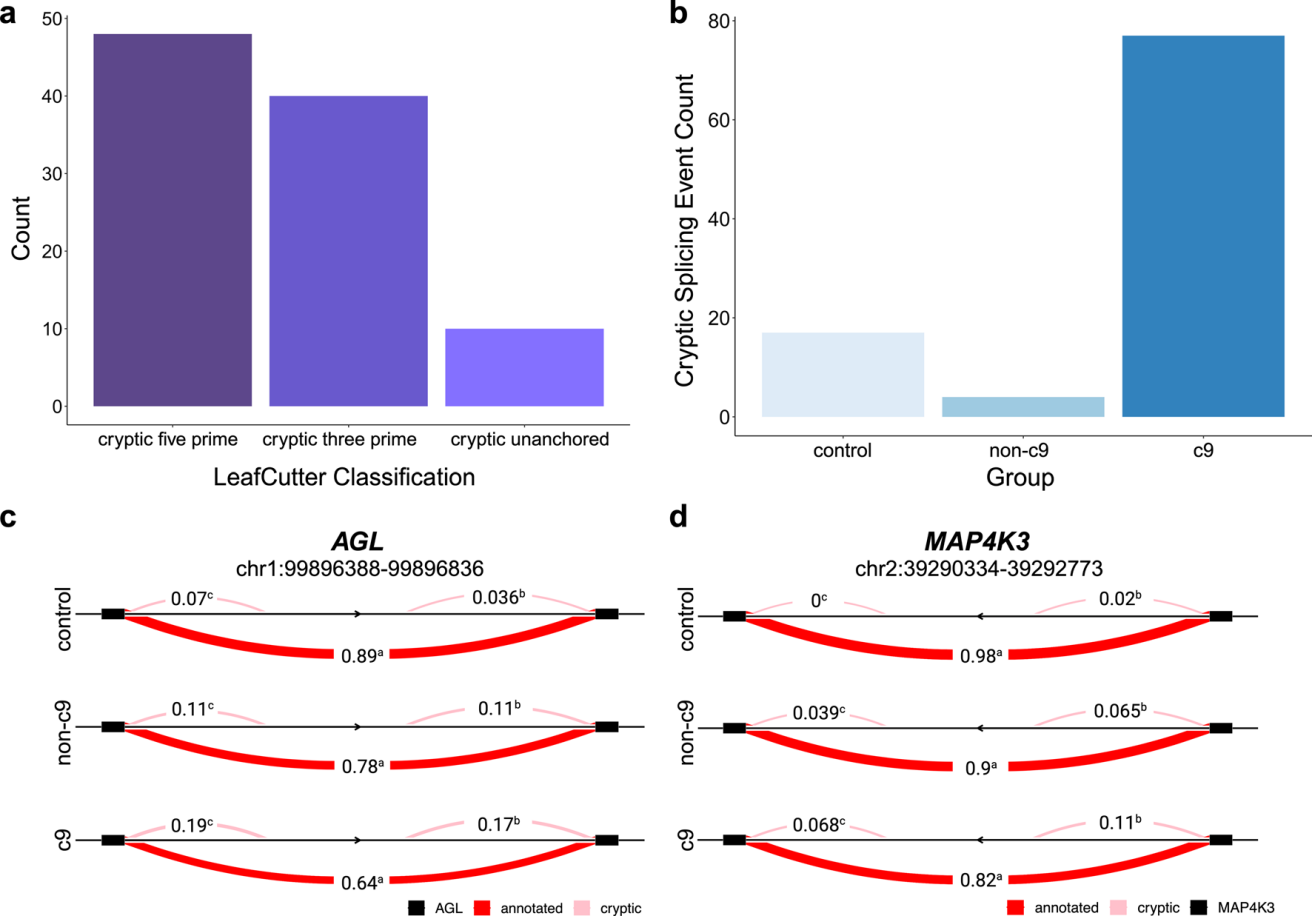
Annotation-based cryptic splicing. **a** Barplot showing number of differential splicing events for the 3 types of splicing events that we considered to be cryptic by annotation identified across all 3 pairwise comparisons. **b** Barplot showing the number of cryptic exons assigned to each group*.* **c** Sashimi plot of *AGL* (left) and *MAP4K3* (right) cryptic exons. These events were identified in both cryptic cassette and cryptic annotation analyses. Pink lines represent cryptic splice junctions as determined by LeafCutter and solid red lines represent annotated splice junctions. Numbers represent the average inclusion for that splice junction. Control = control subjects, non-c9 = patients without a *C9orf72* repeat expansion, c9 = patients with a *C9orf72* repeat expansion

Furthermore, we implemented an annotation-based cryptic splicing approach using LeafCutter output. Cryptic splicing events in this analysis were identified regardless of the PSI in a given group. We included “cryptic five prime”, “cryptic three prime”, and “cryptic unanchored” LeafCutter assignments in our count of cryptic splicing events (Fig. 4a). Overall, we identified 98 cryptic splicing events across all 3 analyses. We first looked at the amount of each type of event present in our samples. In total, there were 48 cryptic five prime splice junctions, 40 cryptic three prime splice junctions, and 10 cryptic unanchored events (Fig. 4a). We next assigned cryptic splicing events to the group that had the highest inclusion of the cryptic junction and looked at the number of these events present in each group. We found 77 cryptic splicing events in the c9 group, 4 cryptic splicing events in the non-c9 group, and 17 cryptic splicing events in the control group (Fig. 4b, Online Resource Table 8).

Two cryptic splicing events were discovered using both the cryptic cassette and annotation-based analyses in c9 patients, which were in the genes *AGL* and *MAP4K3* (Figs. 3b, 4c, d), increasing our confidence that these novel cryptic cassette exons might be real. For *AGL*, this cassette exon was included approximately 28% of the time in c9 patients, 16% of the time in non-c9 patients, and 6% of the time in controls. In *MAP4K3*, this cassette exon was included approximately 13% of the time in c9 patients, 8% of the time in non-c9 patients, and 1% of the time in controls. To validate the presence of these cryptic exons, we completed cDNA sequencing. For each event, we selected 3 individuals with high inclusion of the cryptic exon and 3 individuals with low or no inclusion, based on our RNAseq data. Using cDNA sequencing, we confirmed the presence of these two cryptic cassette exons, validating our results for both *AGL* and *MAP4K3* (Online Resource Fig. 7a, b).

**Fig. 5:**
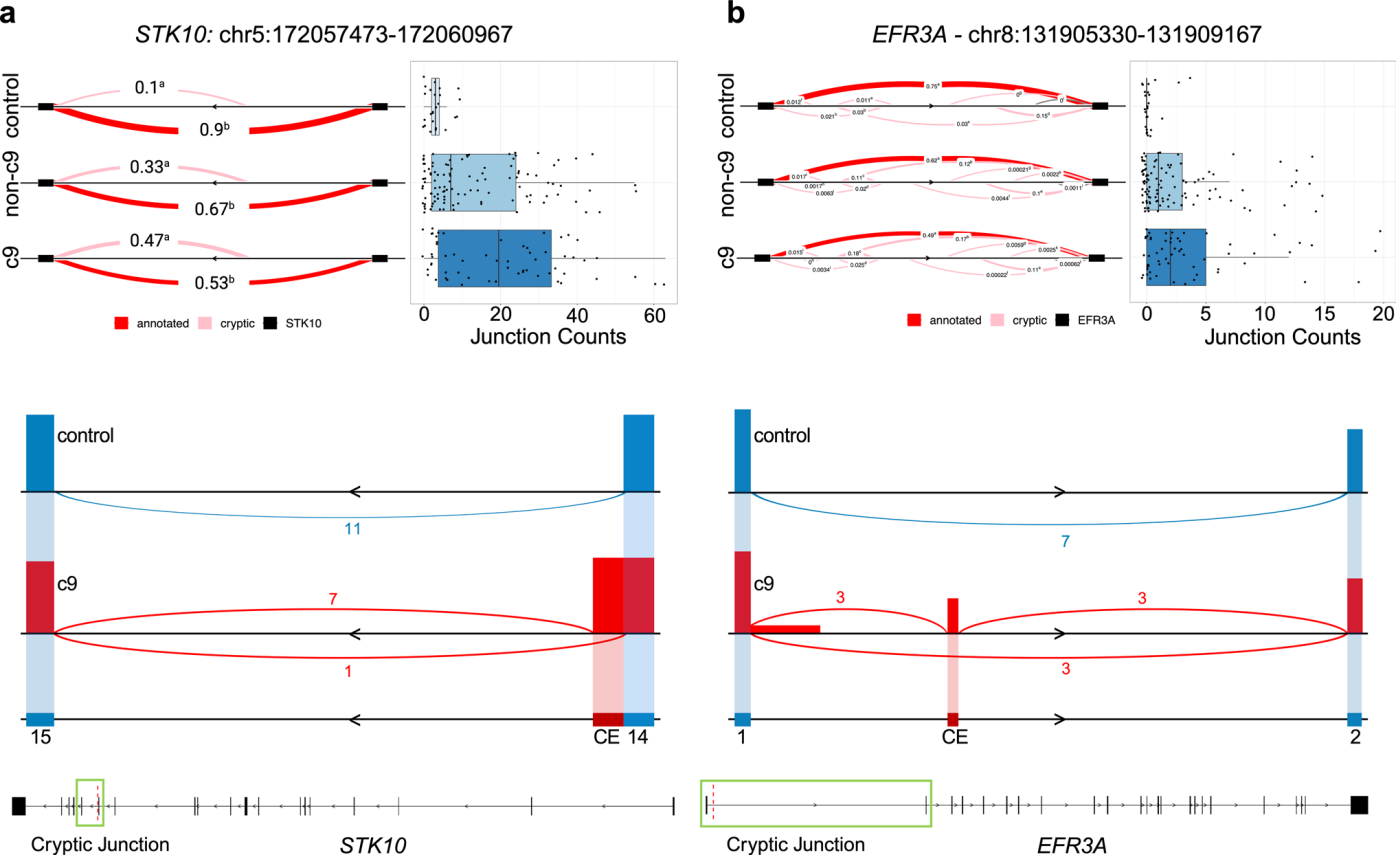
Select cryptic splicing events. **a** *Left:* Sashimi plot of *STK10* cryptic exon. Pink lines represent cryptic splice junctions as determined by LeafCutter and solid red lines represent annotated splice junctions. Numbers represent percent spliced in for that group. *Right:* Boxplot of intron junction counts for the cryptic splicing event shown in the sashimi plot. *Bottom:* Long-read sequencing coverage for 2 samples, 1 c9 patient (red) and 1 control subject (blue). The number represents the number of reads spanning that splice junction. **b** *Left:* Sashimi plot of *EFR3A* cryptic exon. Pink lines represent cryptic splice junctions as determined by LeafCutter and solid red lines represent annotated splice junctions. Numbers represent percent spliced in for that group. *Right:* Boxplot of intron junction counts for the cryptic splicing event shown in the sashimi plot. *Bottom:* Long-read sequencing coverage for 2 samples, 1 c9 patient (red) and 1 control subject (blue). Control = control subjects, non-c9 = patients without a *C9orf72* repeat expansion, c9 = patients with a *C9orf72* repeat expansion. Boxplots represent the median with interquartile range (IQR)

In addition, we selected 2 top cryptic splicing events (*STK10* and *EFR3A*; Fig. 5a, b) from the annotation-based analysis for validation given their significance, relatively large effect size, and decent coverage. To validate these events, we leveraged long-read RNAseq (Iso-Seq) data we recently obtained from the cerebellum for a small number of individuals included in our short-read study (*n* =  2). In *STK10* (c9 vs. control, FDR = 5.14E-07)*,* we showed that the PSI of this cryptic exon was 47% in the c9 group, 33% in the non-c9 group, and 10% in controls. For *EFR3A* (c9 vs. control, FDR = 8.83E-11), the PSI was 18% in the c9 group, 11% in the non-c9 group, and 3% in controls. For the validation, we included 1 c9 patient who had high coverage of both cryptic splicing junctions in our short-read data (63 reads for *STK10* and 13 reads for *EFR3A*) and 1 control subject who had 0 coverage of these splice junctions in our short-read data (Fig. 5a, b). We visualized the splicing events in these 2 individuals using IGV. First, for *STK10*, we confirmed that this event is an extension of the canonical five prime splice junction in exon 14 (Fig. 5a). Comparing the 2 subjects at this splice junction, we found that the individual with inclusion of this splicing event in our short-read data had 7 reads covering the cryptic splice junction and 1 covering the canonical splice junction, whereas in the individual who did not have the cryptic event in the short-read data, we had 0 reads covering the cryptic splice junction and 11 reads covering the canonical splice junction in the long-read data (Fig. 5a). For *EFRA3*, in the c9 patient with high coverage, we found 3 reads mapping to a cryptic exon located between exons 1 and 2, which contained the identical five prime splice junction as observed in the short-read data and a three prime splice junction that was not detected in the short-read data. This was a complex cluster, with 7 unique cryptic splicing junctions in our short-read data, so this exemplified how long-read data may help resolve complex events (Fig. 5b). All 7 reads in our Iso-Seq data in this region mapped to the canonical splice junctions for the control subject.

To demonstrate that we could validate skiptic splicing events too, we selected top skiptic cassette exons, which were present in the genes *CEP170* (c9 vs. control, FDR = 9.02E-12) and *HSPH1* (c9 vs. control, FDR = 7.20E-06), based on their significance, effect size, and coverage. For both events, we detected a high coverage of the skipped exon in our c9 patient and low coverage of the exon skipping in our control subject in the short-read data (Online Resource Fig. 8a). Our long-read data also confirmed these skiptic exons, where in *CEP170* the c9 patient had 7 reads covering the skipped junction, while the control had 0 reads covering the skipped junction, and in *HSPH1* where the c9 patient had 36 reads covering the skipped junction and the control showed 0 reads covering the skipped junction (Online Resource Fig. 8b). This already demonstrates the value of Iso-Seq data, which was able to validate both skiptic and cryptic splicing events that were present in c9 patients, while also helping to unravel some of the complexity that we observed in the short-read splicing data.

**Fig. 6:**
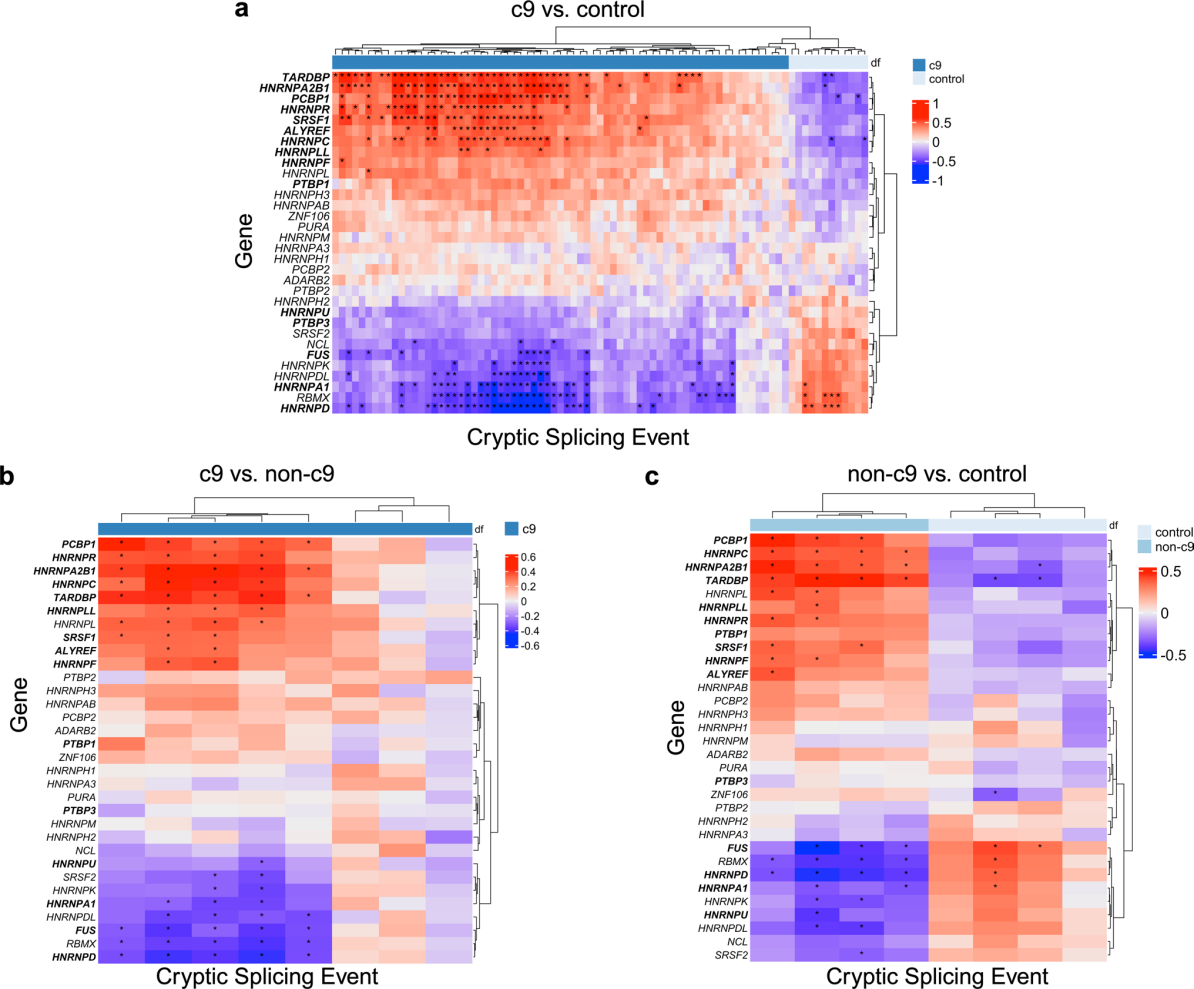
Cryptic splicing correlations. **a**–**c** Correlation heatmaps between cryptic splicing events in the annotation-based analysis and residual gene expression values for select RNA-binding proteins, **a** when comparing *C9orf72* expansion patients to controls (c9 vs. control), **b** *C9orf72* expansion patients to non-*C9orf72* expansion patients (c9 vs. non-c9), and **c** non-*C9orf72* expansion patients to controls (non-c9 vs. control). Colors represent Pearson’s correlation coefficient. Stars indicate significant correlations after correction for multiple testing (Bonferroni *P* Value < 0.05). Differentially expressed genes are bolded

Finally, because aggregated TDP-43 is generally not present in the cerebellum, we examined the expression of *TARDBP*, the gene that encodes TDP-43, and additional genes that encode RNA-binding proteins, including some known to be sequestered by RNA foci [6, 8, 14, 15, 46]. We noticed that 15/32 (47%) were differentially expressed between groups, such as *TARDBP* (FDR = 0.011), *HNRNPA1* (FDR = 0.013), *HNRNPF* (FDR = 0.007), *SRSF1* (FDR = 0.021), and *ALYREF* (FDR = 0.043; see Online Resource Table 2). We then checked if their expression correlated with the inclusion of any of the cryptic splicing events we identified. We, therefore, performed a correlation analysis between the residual expression of selected genes and the junction ratio of the cryptic splicing events. We identified 335, 53, and 64 significant correlations in the c9 vs. control, c9 vs. non-c9, and non-c9 vs. control analyses, respectively, when including annotation-based cryptic events (Fig. 6a–c). Interestingly, we found a consistent pattern, where a select set of genes, which includes *TARDBP*, *HNRNP2AB1*, *SRSF1*, and *PCBP1*, were positively correlated with cryptic splicing event inclusion in c9 patients, and another set of genes, which includes *HNRNPD*, *FUS*, *HNRNPK*, and *HNRNPA1*, were negatively correlated with cryptic splicing event inclusion in c9 patients (Fig. 6a–c). As an example, the *STK10* cryptic splice junction was highly correlated with the RNA expression of several RNA-binding proteins, including a positive correlation with *TARDBP* (c9 vs. control, Bonferroni *P* Value = 2.34E-07, *r *= 0.632) and *HNRNP2AB1* (c9 vs. control, Bonferroni *P* Value = 4.85E-07, *r* = 0.623)*,* and a negative correlation with *FUS* (c9 vs. control, Bonferroni *P* Value = 6.24E-04, *r *= −0.526) and *HNRNPD* (c9 vs. control, Bonferroni *P* Value = 1.68E-08, *r* = −0.660; Online Resource Fig. 9). Other top hits, including cryptic cassette exons as seen in *MAP4K3* and *MTX2*, showed comparable patterns (Online Resource Fig. 10a, b). Notably, these patterns remained similar when not adjusting for cellular composition and using CQN gene expression values (data not shown).

## Discussion

In this study, we describe transcriptomic alterations in the cerebellum of patients with a *C9orf72* repeat expansion. Traditionally, the cerebellum has not been considered a primary affected region in either ALS or FTLD. However, recent studies have suggested that the cerebellum is susceptible to pathological accumulation of RNA foci and DPR proteins that are produced directly from the *C9orf72* repeat expansion [26]. We and others have shown transcriptomic changes in the cerebellum of individuals with ALS and/or FTLD who harbor the *C9orf72* repeat expansion [24, 33, 67, 84]. Consistent with previous results, we observed that *C9orf72* expression is significantly decreased (Fig. 1d) in the cerebellum of c9 patients [84]. Additionally, through differential gene expression analysis and co-expression analysis (DarkRed module), we found that the expression of *HOX* genes is upregulated in the cerebellum of patients with a *C9orf72* repeat expansion, confirming what we demonstrated using a gene expression assay [24]. The *HOX* gene family plays an important role in neuronal development [53, 69]. We hypothesized that the activation of this gene family may be a compensatory mechanism for loss of *C9orf72* expression [24]. Here, we confirm that this upregulation is specific to c9 patients (Fig. 1a, b). Along these lines, we found several interesting modules of co-expressed genes that were associated with c9 status and contained multiple disease-relevant genes. For example, the Blue module, which was enriched for small molecule metabolic processes, was negatively associated with the c9 group when compared to controls and contained 19 disease-associated genes, including *C9orf72*. Moreover, multiple of these c9-associated modules were enriched for RNA processing pathways (Turquoise, Royal Blue). These results provide additional evidence that these molecular pathways have a role in the disease process and suggest that they might be relevant in the cerebellum. In addition to these differential expression and co-expression analyses, our deconvolution analysis revealed a potential reduction of oligodendroglial-lineage cells in individuals with a *C9orf72* repeat expansion. This reduction is of particular interest because studies have previously implicated oligodendrocytes in the ALS/FTLD disease spectrum [22, 23, 34, 82, 87]. Regardless, we acknowledge that our observations are highly preliminary. We realize, for instance, that the marker used for our immunostaining experiment, OLIG2, can stain other cell types and may have a preference for oligodendrocyte precursor cells [54, 62, 86, 89]. As such, further studies are required to clarify if specific oligodendrocyte cell lineage populations (e.g., oligodendrocyte precursor cells, pre-myelinating oligodendrocytes, or myelinating oligodendrocytes) are affected and, if so, how this relates to *C9orf72*-associated diseases.

Additionally, as RNA processing and splicing are key drivers of the ALS/FTLD disease spectrum, we focused on alternative splicing. We found multiple alternative splicing events in genes that have been previously implicated in ALS and/or FTLD (Fig. 2c). This includes an exon skipping event in *CAMTA1,* a gene that contains variants that have been implicated as a clinical modifier of ALS survival time [25]. We also found an exon skipping event in *DCTN1,* which is a gene that harbors variants that confer risk of both ALS and FTLD. Protein levels of DCTN1 have been shown to be reduced in an ALS mouse model and regulate TDP-43 aggregation [18, 43, 58, 85]. In addition, we identified increased inclusion of an annotated exon in *SS18L1,* which has been associated with familial ALS [80]. Finally, we found a complex splicing event located in intron 1 of *C9orf72* (Fig. 2d)*,* where there seems to be an increased presence of a cryptic splicing event in c9 patients (chr9:27,567,164–27,572,766; c9-PSI = 21%, non-c9-PSI = 0.9%). In this cluster, 11 splice junctions were identified. Interestingly, in multiple *C9orf72* transcripts, the repeat expansion is located in intron 1 [84]. More detailed analyses need to be performed to fully understand this complex splicing event.

Previously, alternative splicing of cassette exons was shown to be present at high levels in c9 patients compared to both sporadic ALS patients and controls [67]. We specifically looked at cassette exon splicing in our data and revealed further evidence of dysregulated exon skipping in c9 patients, where we detected a high number of these events in c9 patients compared to both non-c9 patients and controls. We were particularly interested in cryptic splicing as this appears to be a crucial pathogenic mechanism across this disease spectrum [7, 10, 51, 52, 68]. We identified an increased presence of cryptic splicing events in the c9 group compared to both the non-c9 and control groups in our cryptic cassette and annotation-based analyses. Overall, we identified 4 cryptic cassette exons and 77 cryptic splicing events based on annotations in c9 patients (Figs. 3a, 4b). In non-c9 patients, we detected 1 and 4 cryptic events, respectively. Presently, we can only speculate why few cryptic events were found in non-c9 patients. As opposed to our c9 group, it seems plausible that our non-c9 group is more heterogeneous in terms of underlying mechanisms (e.g., due to various genetic and environmental factors), which might hamper our ability to pick relatively rare events up in the cerebellum of non-c9 patients.

Of note, *STK10*, *EFR3A*, and *MAP4K3* had large effect sizes and/or low inclusion in controls. *STK10*, serine-threonine kinase 10, is involved in Rhoc GTPase activity [42], which in the brain has been shown to be crucial for neuronal development [29, 77]. *EFR3A*, Eighty-five Requiring 3A, has been suggested to play an important role in synaptic development in the human fetal brain [59]. Both of these events, like the *HOX* genes, further implicate a role for neuronal development in the cerebellum of patients with the *C9orf72* expansion. Relevant to neurological diseases, rare variants in *EFR3A* have been shown to cause autism and knockout of the gene causes spiral ganglion neurons to degenerate in mouse models [31]. *MAP4K3* is also a serine-threonine kinase that plays an important role in the TFEB pathway [12], in turn functioning as protein kinase, with other suggested roles in cell death and autophagy [72]. Genes of the *MAPK* family have been implicated in ALS generally as they play important roles in cell proliferation and survival. Notably, multiple inhibitors of the MAPK pathway have been tested in various models of ALS, including those targeting MAP4K, which have been shown to increase motor neuron survival time in *SOD1* and mutant TDP-43 induced pluripotent stem cell lines [90]. Based on our current analyses, these events could be further studied to determine the functional consequences of these splicing events and how they may contribute to c9-related diseases. In addition, it is important to consider that these events may only be present in specific cell types. Assessing cryptic splicing may be a challenge in single-cell or single-nuclei RNAseq data; however, recently one group completed a single-cell analysis of the *STMN2* and *KALRN* cryptic exons in the context of *C9orf72* and identified specific vulnerable cell types [28]. Future studies, similar to our present study, will need to be performed to identify more of these potentially pathogenic cryptic splicing events.

Though we know that the cerebellum is primarily spared from TDP-43 pathology, TDP-43 protein levels may be reduced in FTLD-TDP patients in this region [63]. The cerebellum does, however, contain other pathological inclusions in c9 patients that are ubiquitin and p62 positive [1, 64, 81], in addition to RNA foci and DPR proteins produced from the repeat expansion. Some of these c9-specific pathologies colocalize with and sequester RNA-binding proteins in the cerebellum [14, 26], including hnRNPs, which have been shown to play a role in suppressing cryptic exons, such as hnRNP K [7, 75]. We hypothesized that this could explain the increase in cryptic exons in c9 patients but not non-c9 patients. Therefore, we completed correlations between the expression of genes that encode RNA-binding proteins and our cryptic splicing events, which demonstrated that at least some of these cryptic events are associated with the expression of various genes that encode RNA-binding proteins. This aligns with the fact that several cryptic splicing events we detected (e.g., in *TPCN1* and *SNHG14*) are in genes known to be regulated by RNA-binding proteins like hnRNP K [7].

Notably, we detected primarily positive correlations between *TARDBP* and c9 cryptic exon junctions. When looking at our differential expression data, we did show that *TARDBP* was differentially expressed, with increased RNA expression in c9 patients, contrary to what has been shown at the protein level. TDP-43 autoregulation is well established [4], and therefore, it may not be surprising that we saw increased expression levels in response to previously reported decreased protein levels [63]. Overall, there appeared to be 2 primary clusters of genes that were either consistently positively or negatively correlated with cryptic exon inclusion. In addition to *TARDBP*, this includes genes like *HNRNPA2B1*, *FUS*, *SRSF1*, and *ALYREF* (Fig. 6a–c)*.* While further study is needed to evaluate the specific effects of each of the proteins that these genes encode for, our data does suggest that these non-TDP-43 RNA-binding proteins may also play an important role in splicing alterations. One should be mindful, however, of the fact that we examined RNA expression-based correlations of select RNA-binding proteins, which, as demonstrated by TDP-43, are not necessarily indicative of protein levels or RNA-binding protein pathology. Additionally, we would like to stress that future large-scale studies that integrate RNA foci burden and/or DPR protein levels should be performed to specifically investigate the direct role of these pathological features on altered splicing.

In the current study, we performed a transcriptome-wide comparison of differentially expressed genes and WGCNA modules to the frontal cortex, a primary affected region. Importantly, many relevant genes (e.g., *C9orf72* and *TARDBP*) as well as pathways (e.g., small molecule metabolic processes and protein modification) were identified in both regions. This demonstrates that we can capture vital biological changes in the cerebellum that are observed in primary affected regions, like the frontal cortex, while also capturing unique transcriptomic alterations (e.g., *HOX* genes). Additional studies have previously characterized the transcriptome of primary affected brain regions in patients with a *C9orf72* repeat expansion [33, 67]. In line with our findings, these studies detected decreased levels of *C9orf72* in patients with a *C9orf72* expansion, while no differences *HOX* genes were reported [33, 67]. Interestingly, evaluation of alternative splicing specifically in *C9orf72* patient brain tissue suggested that splicing alterations were more profound in the cerebellum compared to the frontal cortex [67]. More well-designed, large-scale transcriptomic studies of *C9orf72* patients are needed to allow in-depth comparisons across brain regions, which may reveal critical similarities and differences.

Our study raises many additional questions that require more investigation. Newer sequencing technologies, such as long-read sequencing and single-cell/nuclei sequencing should be capable of addressing some of the questions. Specifically, long-read sequencing technologies could aid in identifying and/or interpretating alternative splicing events, especially complex events, like the ones in *EFR3A* or *C9orf72*. This technology is a powerful way to discover disease-relevant transcript variants and cryptic splicing events that may not be detectable with short-read sequencing. At the same time, single-cell/nuclei studies can assist in increasing our understanding of gene expression, splicing, and cellular proportion differences. For example, while our bulk RNAseq data has allowed us to estimate changes in some cell types, this approach has a limited ability to comprehensively interrogate cell-type-specific gene expression and splicing changes. As others have shown, unique cell types respond differently to pathology [76]. Future single-cell/nuclei studies in the cerebellum will be essential for revealing cell-type-specific disease mechanisms and vulnerable cell populations, similar to a recent study of the frontal cortex [47]. This would help to determine whether a given event is mainly present in a specific type of cell and to elucidate whether differences in cellular proportions might, in part, contribute to some of the observed findings (e.g., splicing events). Combining these technologies (long-read + single-cell/nuclei sequencing) could facilitate the prioritization of disease-relevant splicing events and cell-specific alterations.

To conclude, we discovered abundant transcriptomic changes in the cerebellum of patients with a *C9orf72* repeat expansion. Analysis of gene expression emphasized a role for genes involved in neuron development and RNA processing. Additionally, we detected an increase in cryptic exons in the cerebellum of c9 patients, which may be due to c9-specific pathologies in the cerebellum, possibly impacting various RNA-binding proteins. Overall, this study further emphasizes a role for the cerebellum in c9-related diseases and that c9-specific pathologies may contribute to cryptic splicing, particularly in neuroanatomical regions without characteristic TDP-43 pathology.

### Supplementary Information

Below is the link to the electronic supplementary material.

Supplementary file1 (PDF 15211 kb)

Supplementary file2 (XLSX 4443 kb)

## Data Availability

Illumina short-read RNAseq data and corresponding sample metadata has been deposited in dbGAP: https://www.ncbi.nlm.nih.gov/projects/gap/cgi-bin/study.cgi?study_id=phs003065.v1.p1 Additional information is available upon reasonable request.

## Abbreviations

AGL: Amylo-alpha-1, 6-glucosidase, 4-alpha-glucanotransferase
ALS: Amyotrophic lateral sclerosis
Bicor: Biweight midcorrelation
c9: Patient with the *C9orf72* repeat expansion
C9orf72: C9orf72-SMCR8 complex subunit
CAMTA1: Calmodulin binding transcription activator 1
Ccs: Circular consensus sequence
CDH22: Cadherin 22
CEP170: Centrosomal protein 170
CHMP2B: Charged multivesicular body protein 2B
CQN: Conditional quantile normalization
DCTN1: Dynactin subunit 1
DPR: Dipeptide repeat
EFR3A: EFR3 homolog A
EPHA4A: EPH receptor A4
FDR: False discovery rate
FTD: Frontotemporal dementia
FTLD-TDP: Frontotemporal lobar degeneration with TDP-43 pathology
FTLD: Frontotemporal lobar degeneration
FUS: FUS RNA binding protein
GRN: Granulin precursor
HNRNPA2B1: Heterogeneous nuclear ribonucleoprotein A2/B1
HNRNPC: Heterogeneous nuclear ribonucleoprotein C
HNRNPD: Heterogeneous nuclear ribonucleoprotein D
HNRNPK: Heterogeneous nuclear ribonucleoprotein K
HOX: Homeobox
HSPH1: Heat shock protein family H (Hsp110) member 1
IGV: Integrative genomics viewer
IQR: Interquartile range
KALRN: Kalirin RhoGEF kinase
MAP4K3: Mitogen-activated protein kinase kinase kinase kinase 3
MAPT: Microtubule associated protein tau
MM: Module membership
MsigDB: Molecular signatures database
NEK1: NIMA related kinase 1
non-c9: Patient without the *C9orf72* repeat expansion
OPTN: Optineurin
PacBio: Pacific biosciences
PCA: Principal component analysis
PCBP1: Poly(RC) binding protein 1
PSI: Percent spliced in
RBMX: RNA binding motif protein X-linked
RIN: RNA integrity number
RNAseq: RNA sequencing
RPKM: Reads per kilobase million
SHOX2: SHOX homeobox 2
SMRT: Single-molecule real-time
SOD1: Superoxide dismutase 1
SOV: Source of variation
SS18L1: SS18L1 subunit of BAF chromatin remodeling complex
STK10: Serine/threonine kinase 10
STMN2: Stathmin 2
TARDBP: TAR DNA binding protein
TBK1: TANK binding kinase 1
TDP-43: TAR DNA-binding protein 43
TFEB: Transcription factor EB
TMEM106B: Transmembrane protein 106B
UNC13A: Unc-13 homolog A
WGCNA: Weighted gene co-expression network analysis
