# Supplementary material for: Abundant transcriptomic alterations in the human cerebellum of patients with a C9orf72 repeat expansion
Source: Acta Neuropathol. 2024 Apr 19;147(1):73. doi: 10.1007/s00401-024-02720-2 (PMC11031479; doi:10.1007/s00401-024-02720-2)

Abundant transcriptomic alterations in the human cerebellum of patients with a *C9orf72* repeat expansion

Authors: Evan Udine[1,2], Mariely DeJesus-Hernandez[1], Shulan Tian[3], Sofia Pereira das Neves[1], Richard Crook[1], NiCole A. Finch[1], Matthew C. Baker[1], Cyril Pottier[1], Neill R. Graff-Radford[4], Bradley F. Boeve[5], Ronald C. Petersen[5], David S. Knopman[5], Keith A. Josephs[5], Björn Oskarsson[4], Sandro Da Mesquita[1,2], Leonard Petrucelli[1,2], Tania F. Gendron[1,2], Dennis W. Dickson[1,2], Rosa Rademakers[1,6,7], Marka van Blitterswijk[1,2*]

Affiliations: [1] Department of Neuroscience, Mayo Clinic, Jacksonville, FL, 32224, USA, [2] Neuroscience Ph.D. Program, Mayo Clinic Graduate School of Biomedical Sciences, Mayo Clinic, Jacksonville, FL, 32224, USA, [3] Department of Quantitative Health Sciences, Mayo Clinic, Rochester, MN, 55905, USA, [4] Department of Neurology, Mayo Clinic, Jacksonville, FL, 32224, USA, [5] Department of Neurology, Rochester, MN, 55905, USA, [6] VIB Center for Molecular Neurology, VIB, Antwerp, Belgium, [7] Department of Biomedical Sciences, University of Antwerp, Antwerp, Belgium

Corresponding Author: Marka van Blitterswijk, M.D., Ph.D., Department of Neuroscience, Mayo Clinic, 4500 San Pablo Rd S, Jacksonville, FL 32224, USA. Telephone: +1 904-953-2226. Email: VanBlitterswijk.Marka@mayo.edu

Contents: Supplementary Online Resource Table and Figure Legends; Online Resource Figures 1-10

Online Resource Table and Figure Legends

Online Resource Table 1. (**a**) List of 50 marker genes used in differential expression analysis. We adjusted for 5 cell types: neurons, microglia, astrocytes, oligodendrocytes, and endothelial cells.

Online Resource Table 2. Differential gene expression summary statistics for each gene tested. The P-ValueGroup and FDRGroup columns represent the ANOVA completed across all three groups. Subsequent columns represent the statistics calculated in the pairwise comparisons. We have included PValue, FDR (Benjamini-Hochberg), and FoldChange values for the pairwise comparisons. *control = control subjects, non-c9 = patients without a C9orf72 repeat expansion, c9 = patients with a C9orf72 repeat expansion.*

Online Resource Table 3. Significant pathway enrichment results for the differential expression analyses. The comparison indicates the pairwise comparison, the Pathway (GOBP) represents the significantly enriched pathway and FDR represents the corrected P-Value (Benjamini-Hochberg). Only significant enrichments for the top 50 genes in each comparison are presented. *control = control subjects, non-c9 = patients without a C9orf72 repeat expansion, c9 = patients with a C9orf72 repeat expansion.*

Online Resource Table 4. WGCNA results for each pairwise comparison including (**a**) c9 vs. control, (**b**) c9 vs. non-c9, and (**c**) non-c9 vs. control. The primary assigned module for each gene is shown. *control = control subjects, non-c9 = patients without a C9orf72 repeat expansion, c9 = patients with a C9orf72 repeat expansion.*

Online Resource Table 5. Differential splicing summary statistics per cluster for (**a**) c9 vs. control, (**b**) c9 vs. non-c9, and (**c**) non-c9 vs. control. *control = control subjects, non-c9 = patients without a C9orf72 repeat expansion, c9 = patients with a C9orf72 repeat expansion.*

Online Resource Table 6. Significant pathway enrichment results for the differential splicing analyses. The comparison indicates the pairwise comparison, the Pathway (GOBP) represents the significantly enriched pathway and FDR represents the corrected P-Value (Benjamini-Hochberg). *control = control subjects, non-c9 = patients without a C9orf72 repeat expansion, c9 = patients with a C9orf72 repeat expansion.*

Online Resource Table 7. Differential cassette exon splicing summary statistics per cluster for (**a**) c9 vs. control, (**b**) c9 vs. non-c9, and (**c**) non-c9 vs. control. *control = control subjects, non-c9 = patients without a C9orf72 repeat expansion, c9 = patients with a C9orf72 repeat expansion.*

Online Resource Table 8. Cryptic splicing by annotation summary statistics per event. The group represents the group with higher inclusion of the cryptic splicing event. *control = control subjects, non-c9 = patients without a C9orf72 repeat expansion, c9 = patients with a C9orf72 repeat expansion.*

Online Resource Fig. 1. Differential gene expression. (**a**) Principal component analysis (PCA) of residual expression values (including cellular composition) for all expressed genes. Each dot represents a sample and colors correspond to the disease group. PC1 explains 17% of all variation and PC2 explains 8%. (**b**) Venn Diagram displaying overlap of differentially expressed genes in all analyses. (**c**) Heatmap of top 50 differentially expressed genes between (*left*) c9 patients and controls, (*center*) c9 patients and non-c9 patients, and (*right*) non-c9 patients and controls. (**d)** Venn diagram displaying the number of overlapping differentially expressed genes in the cerebellum vs. the frontal cortex. c*ontrol = control subjects, non-c9 = patients without a C9orf72 repeat expansion, c9 = patients with a C9orf72 repeat expansion.*

Online Resource Fig. 2. Deconvolution and immunostaining. (**a**) Boxplots of estimated cell proportions from deconvolution analysis. (**b**) Representative images of cerebellar tissue stained for DAPI (blue) and OLIG2 (red). (**c**) Boxplot displaying the average counts of OLIG2+ cells/mm^2 between the 2 investigators. The oligodendrocyte group represents the group as determined by deconvolution analysis (HIGH = high proportion of oligodendrocytes in deconvolution analysis; LOW = low proportion of oligodendrocytes in deconvolution analysis). P-Values were determined using a Wilcoxon rank-sum test (P-Values: NS > 0.05; * < 0.05, ** < 0.01; *** < 0.001, **** < 0.0001). Boxplots represent the median with interquartile range (IQR).

Online Resource Fig. 3. Module trait associations. Heatmap of associations between modules for the (**a**) c9 vs. control analysis, (**b**) c9 vs. non-c9 analysis, and (**c**) non-c9 vs. control analysis. Plots have been generated with weighted gene co-expression network analysis (WGCNA). Each module has been assigned a unique color. Correlations are represented by the color and are shown as the top number in the heatmap and P-Values are shown in parentheses below. Correlations are presented for variables of interest, including the disease group (c9, non-c9, and/or control), neurons, microglia, astrocytes, oligodendrocytes, endothelial cells, gene counts, RNA integrity number (RIN), age at death, sex, and plate. Correlations were completed with adjustment for cell-type-specific markers. *control = control subjects, non-c9 = patients without a C9orf72 repeat expansion, c9 = patients with a C9orf72 repeat expansion.*

Online Resource Fig. 4. Select WGCNA modules. (**a**) The Blue module (c9 vs. control comparison) is shown. Node color represents the primary module assignment. Node size represents the module membership. Edge thickness represents the strength of the interaction. (**b**) Heatmap of residual expression of genes in the Blue module. The colors of the bar at the top refer to the group of each individual. The barplot at the bottom represents the eigengene value for each sample, which is decreased in c9 patients compared to control subjects. (**c**) The DarkRed module (c9 vs. control comparison) is shown. (**d**) Principal component (PC) analysis of residual expression values for the genes included in the DarkRed module. PC1 explains 14% of the variation and PC2 explains 6%. *control = control subjects, non-c9 = patients without a C9orf72 repeat expansion, c9 = patients with a C9orf72 repeat expansion.*

Online Resource Fig. 5. Module preservation plot of preservation scores for cerebellar modules in the frontal cortex for (**a**) c9 vs. control, (**b**) c9 vs. non-c9, and (**c**) non-c9 vs. control. Colors represent the associated module color in the cerebellum. *control = control subjects, non-c9 = patients without a C9orf72 repeat expansion, c9 = patients with a C9orf72 repeat expansion.*

Online Resource Fig. 6. Differential splicing of ALS/FTLD-related genes. Sashimi plots generated in LeafCutter for (**a**) *CAMTA1,* (**b**) *DCTN1,* and (**c**) *SS18L1.* Pink lines represent cryptic splice junctions as determined by LeafCutter and solid red lines represent annotated splice junctions. Numbers represent the average inclusion for that splice junction. *control = control subjects, non-c9 = patients without a C9orf72 repeat expansion, c9 = patients with a C9orf72 repeat expansion.*

Online Resource Fig. 7. Sequencher© (Gene Codes Corporation) software screenshots of exon boundaries (**a**) 25/26 of *AGL* and (**b**) 18/19 of *MAP4K3*. PCR products were amplified from gene specific primer isolated cDNA and sequenced in both directions (the forward reads are shown). Exon boundaries are indicated by blue arrows. The upper chromatogram (cryptic) of each alignment confirms the start and end of the cryptic exon sequence and the lower chromatogram (canonical) shows the canonical exon sequence.

Online Resource Fig. 8. Select skiptic splicing events. (**a**) *Left:* Sashimi plot of *CEP170* skiptic exon. Number represents percent spliced in for that group. *Right:* Boxplot of intron junction counts for the cryptic splicing event shown in the sashimi plot. *Bottom:* Long-read sequencing coverage for 2 samples, 1 c9 patient (red) and 1 control subject (blue). Number represents the number of reads spanning that splice junction. (**b**) *Left:* Sashimi plot of *HSPH1* skiptic exon. Number represents percent spliced in for that group. *Right:* Boxplot of intron junction counts for the cryptic splicing event shown in the sashimi plot. *Bottom:* Long-read sequencing coverage for 2 individuals, 1 c9 patient (red) and 1 control subject (blue). *control = control subjects, non-c9 = patients without a C9orf72 repeat expansion, c9 = patients with a C9orf72 repeat expansion.*

Online Resource Fig. 9. *STK10* cryptic splicing event correlations. Scatter plot of the percent spliced in (PSI) of the *STK10* cryptic splicing event and residual gene expression for select RNA-binding proteins, namely *TARDBP* (Bonferroni P-Value = 1.83E-07, *r* = 0.632), *HNRNPA2B1* (Bonferroni P-Value = 3.79E-07, *r* = 0.623), *HNRNPD* (Bonferroni P-Value = 1.31E-08, *r* = -0.660), and *FUS* (Bonferroni P-Value = 4.87E-04, *r* = -0.526). Correlation coefficients were calculated using a Pearson's correlation within patients with a *C9orf72* repeat expansion and control subjects.

Online Resource Fig. 10. Cryptic cassette exon correlations. (**a-b**) Correlation heatmaps between cryptic cassette exons and residual gene expression values for select RNA-binding proteins, (**a**) when comparing patients with a *C9orf72* expansion to controls (c9 vs. control) and (**b**) patients without a *C9orf72* expansion to controls (non-c9 vs. control). Colors represent Pearson's correlation coefficient. Stars indicate significant correlations after correction for multiple testing (Bonferroni P-Value < 0.05). Differentially expressed genes are bolded.

Online Resource Figure 1

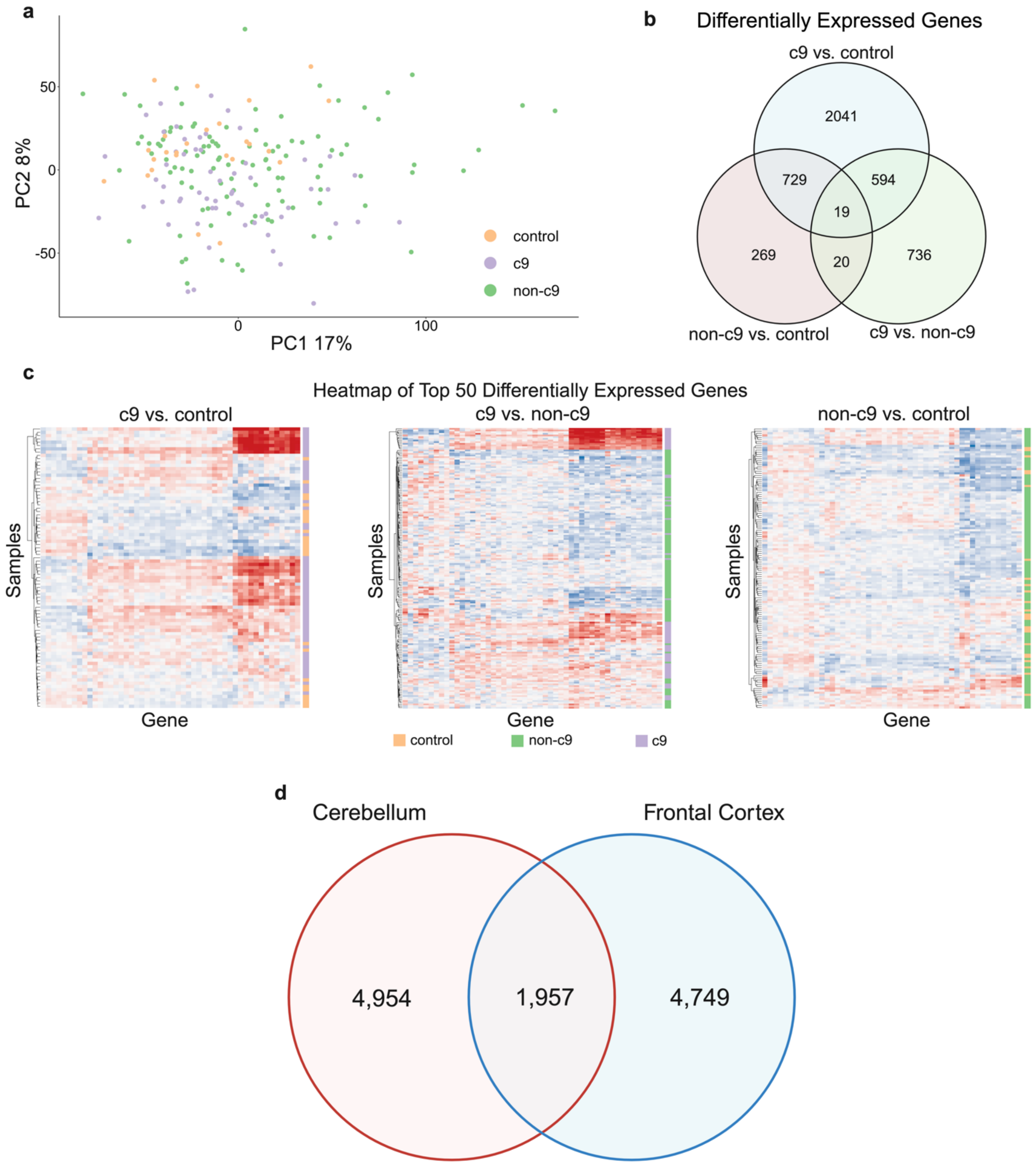

Online Resource Figure 2

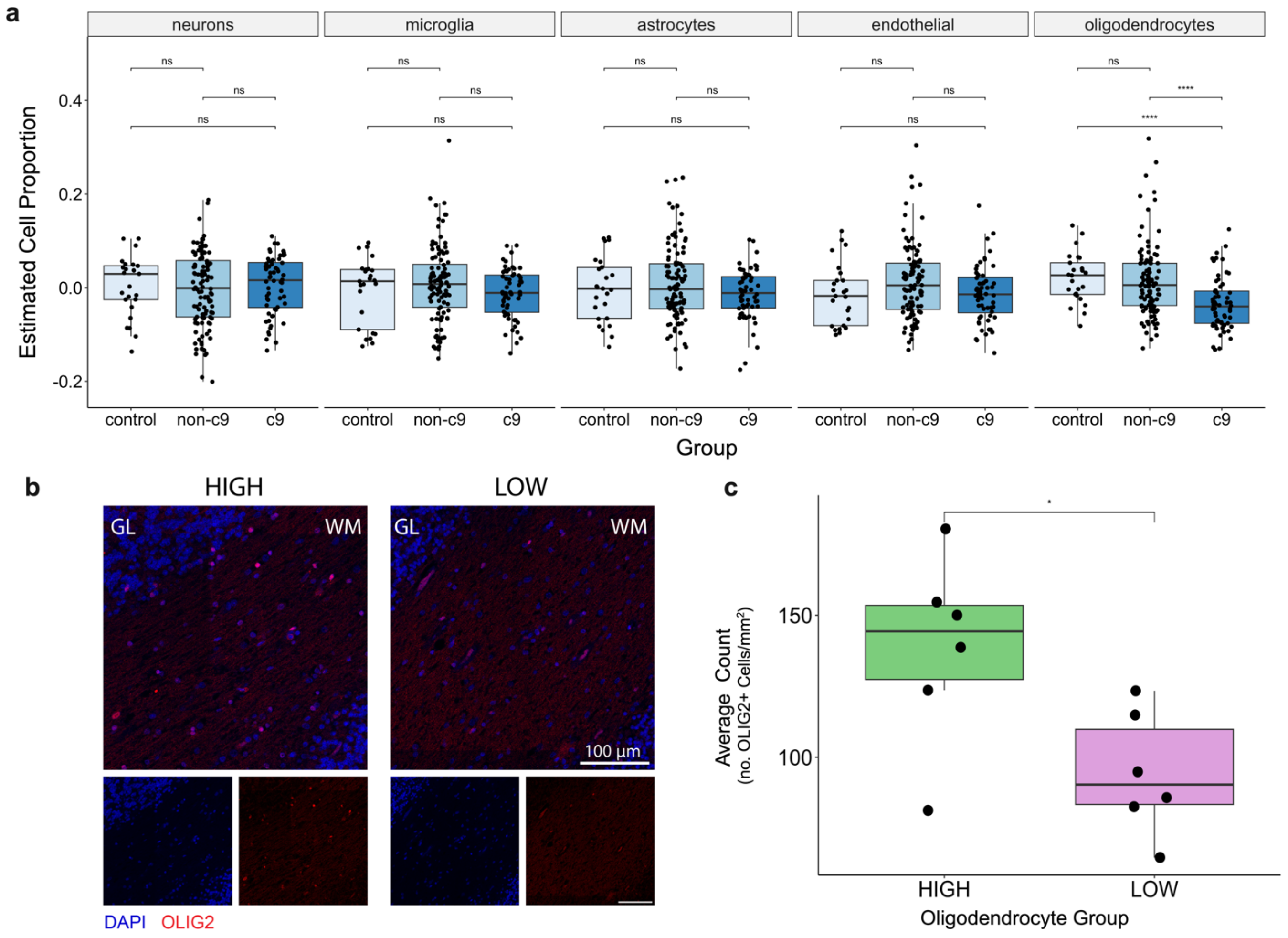

Online Resource Figure 3

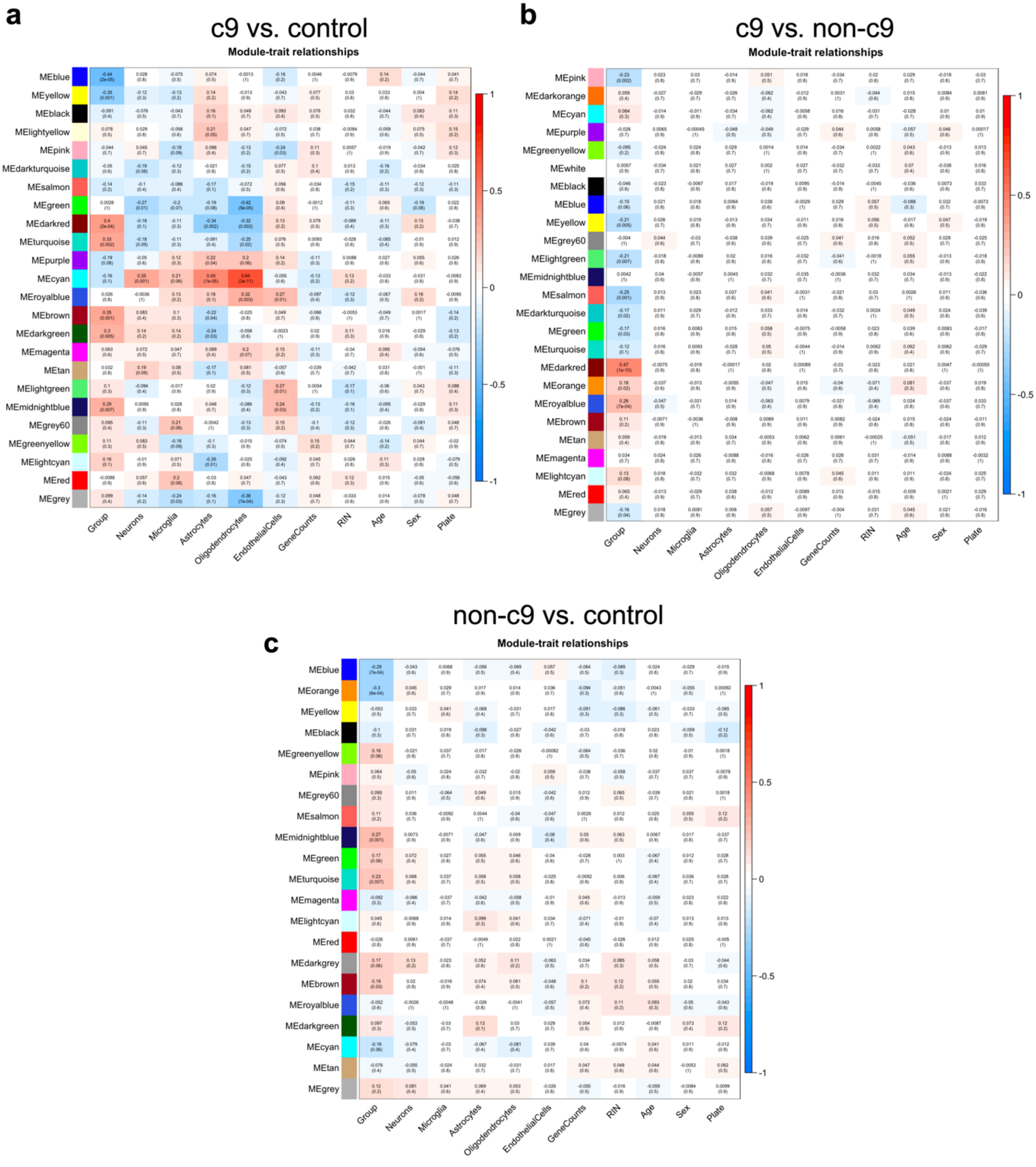

Online Resource Figure 4

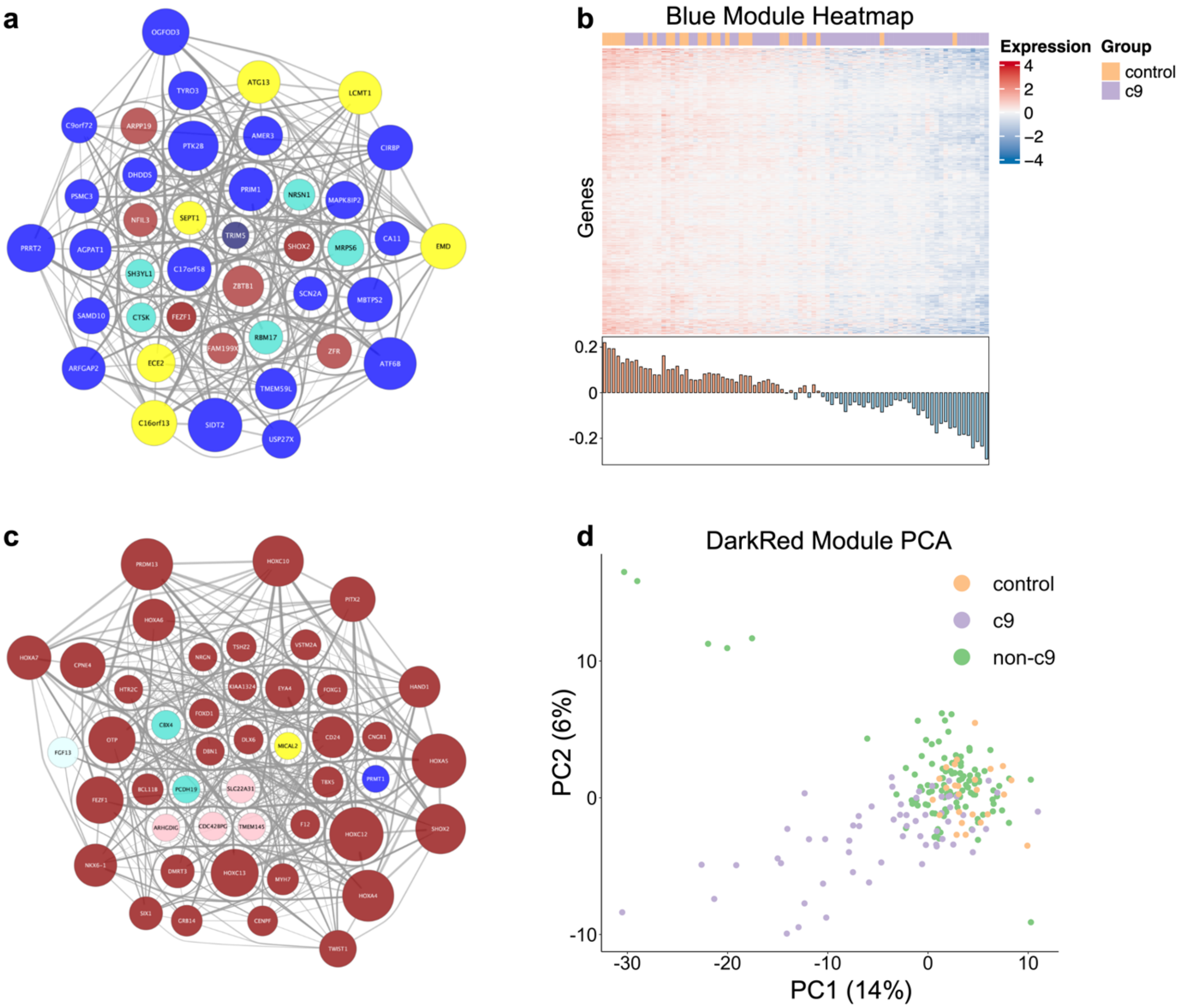

Online Resource Figure 5

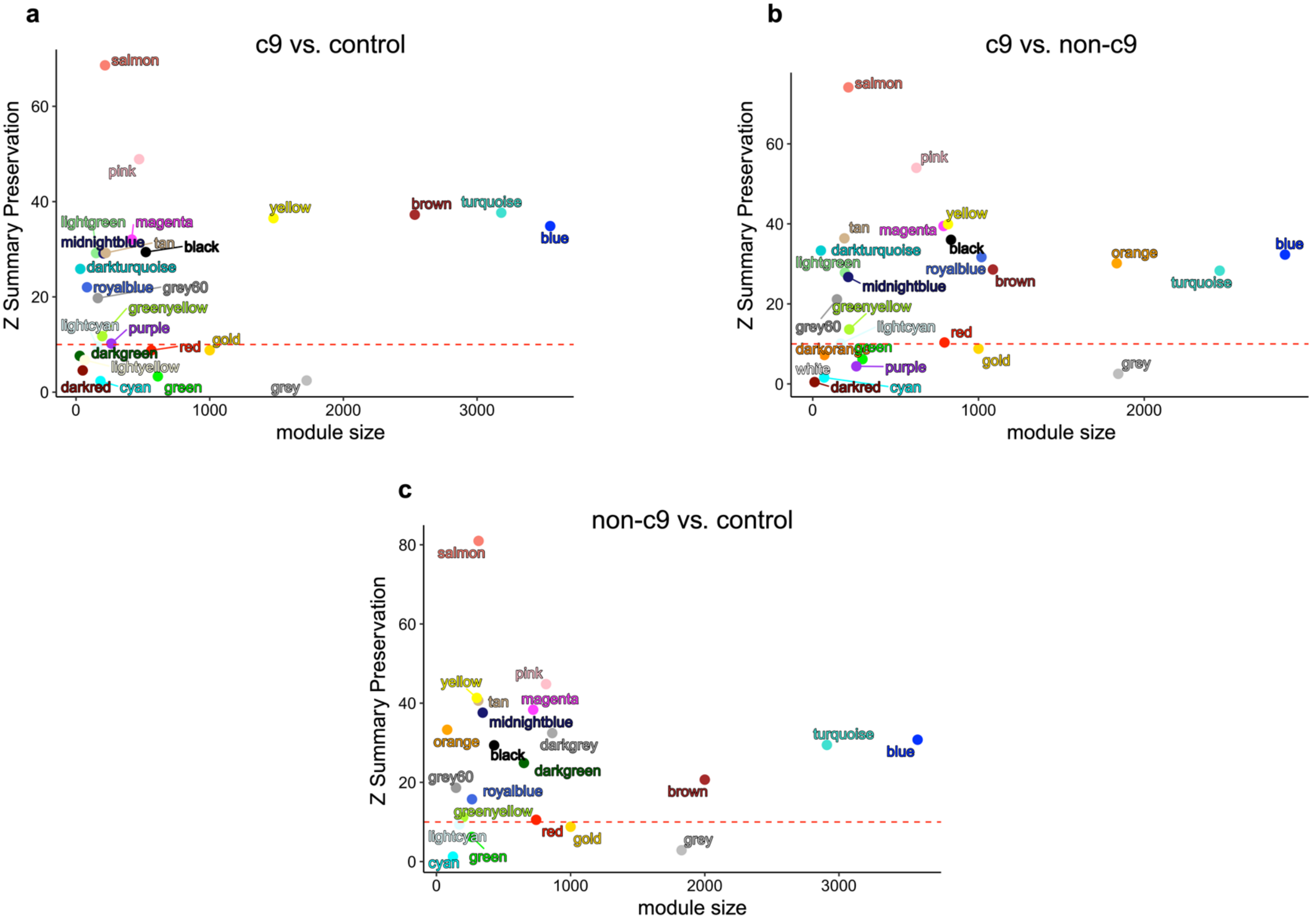

Online Resource Figure 6

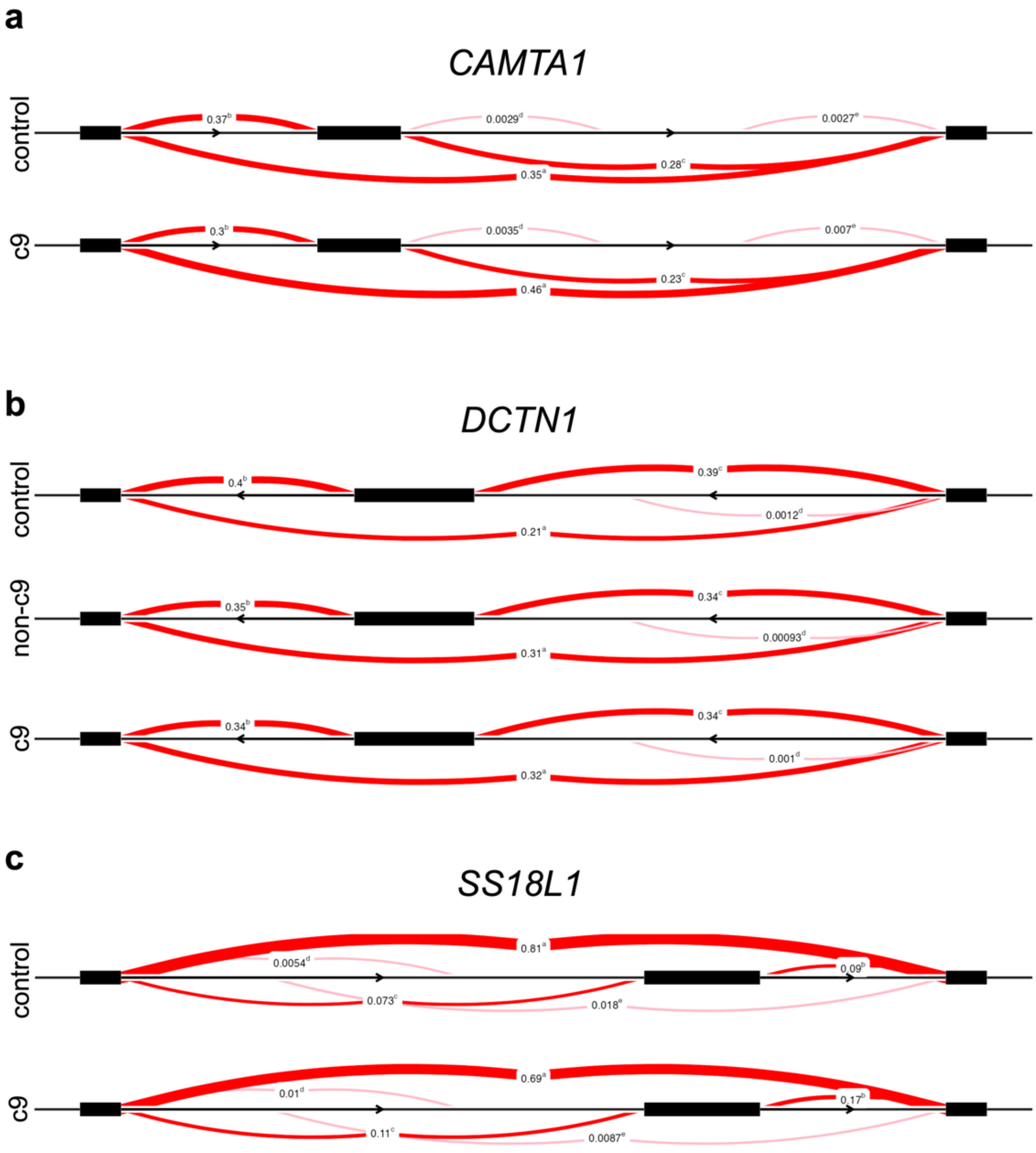

Online Resource Figure 7

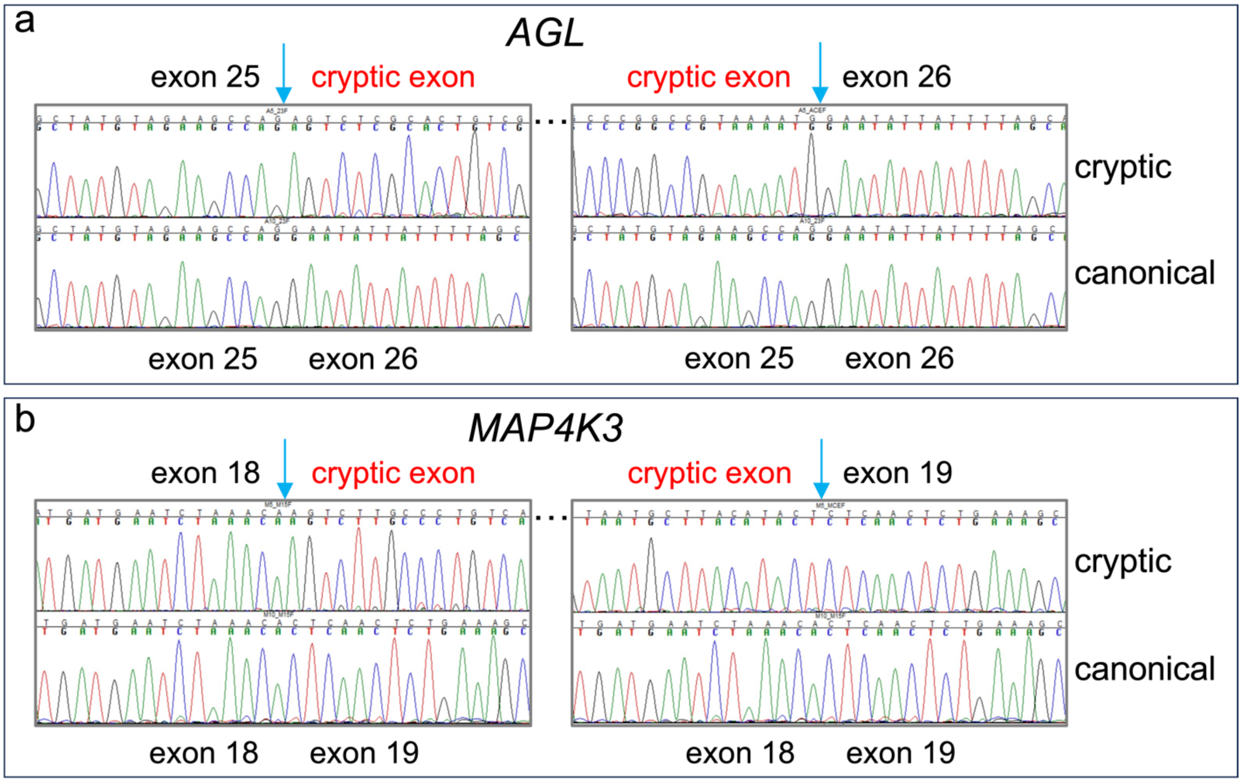

Online Resource Figure 8

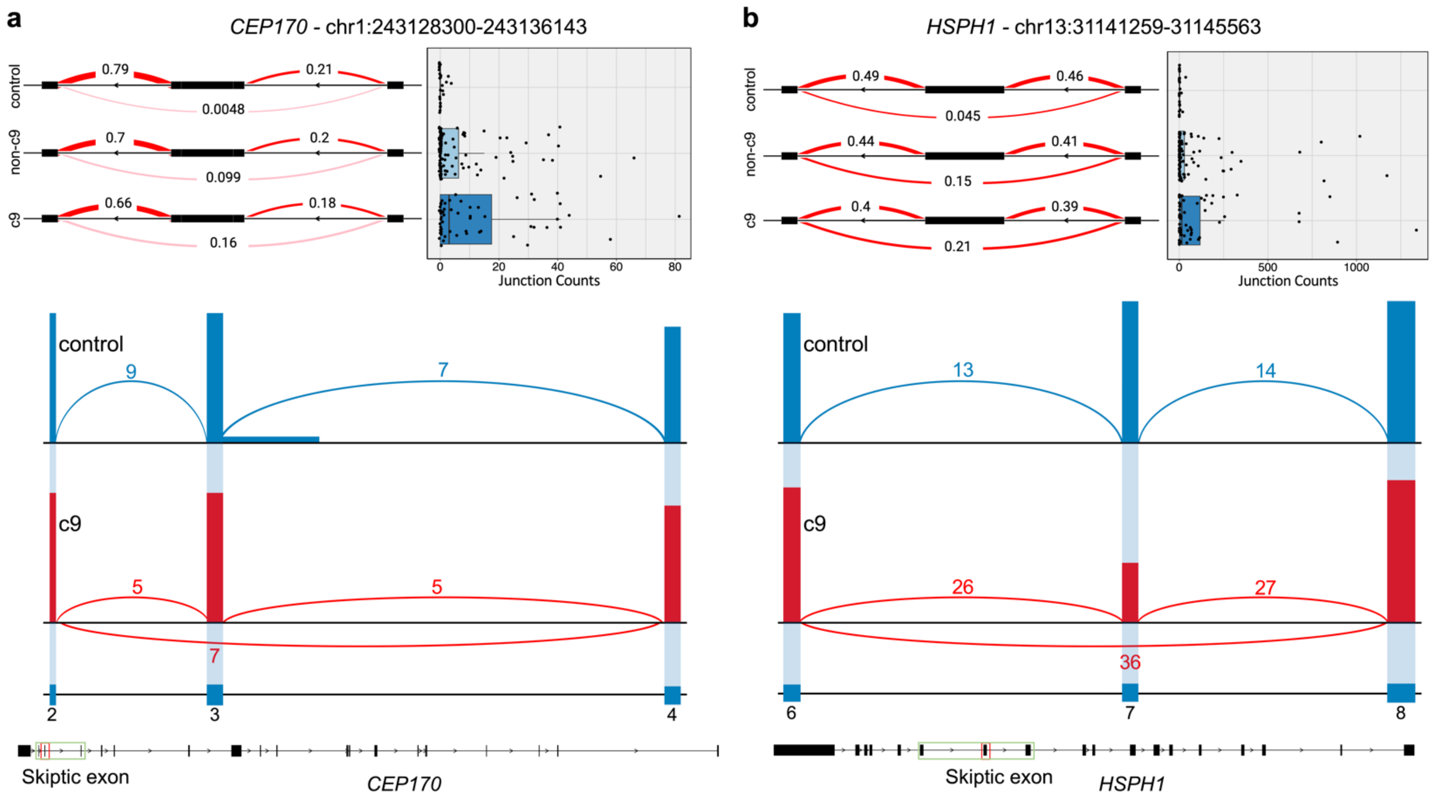

Online Resource Figure 9

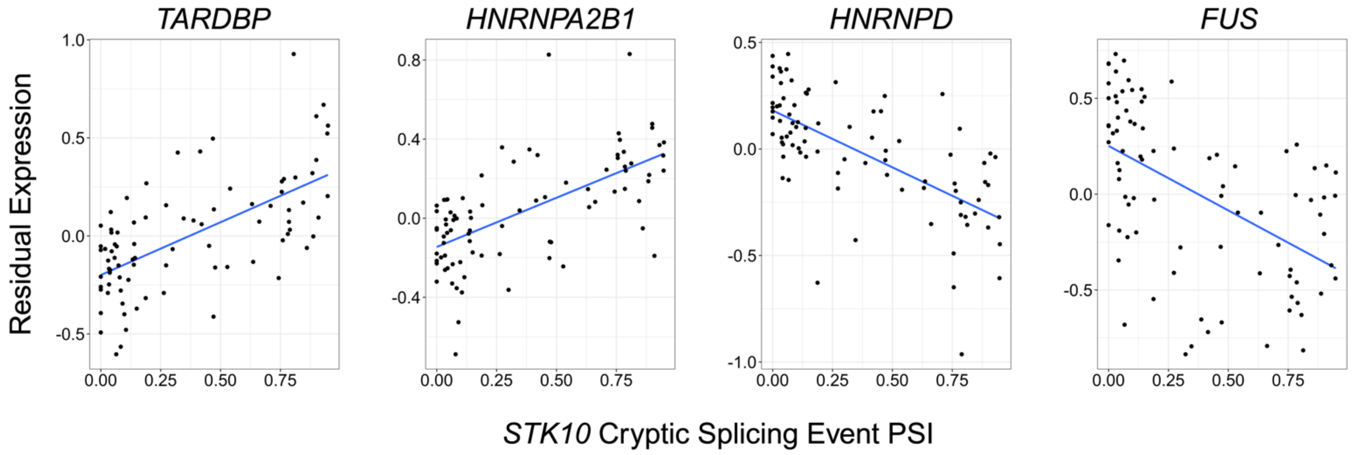

Online Resource Figure 10

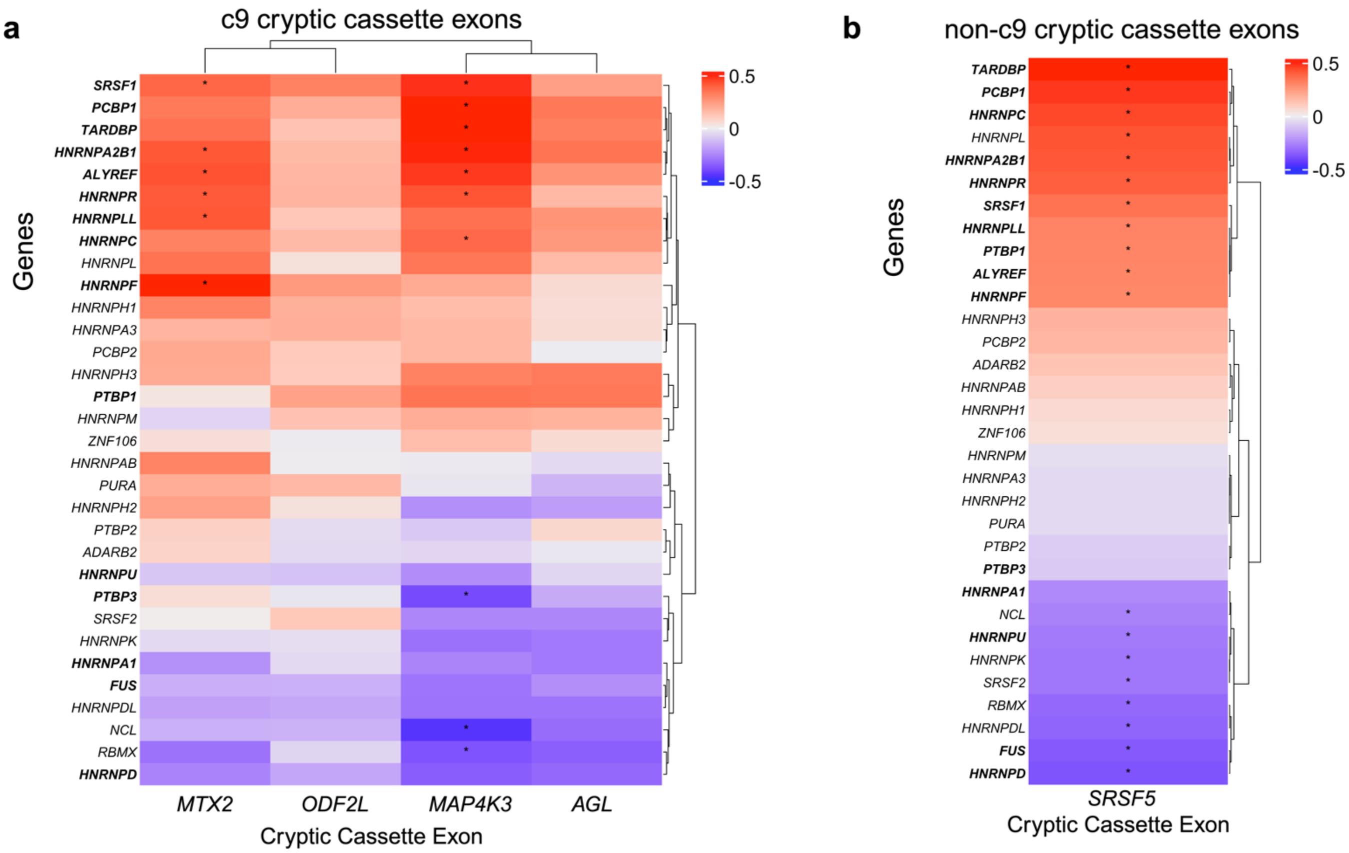

Supplement: Supplementary file 1 — Supplementary file1 (PDF 15211 kb) [file 401_2024_2720_MOESM1_ESM.pdf]
